# Delving into the significance of the His289Tyr single-nucleotide polymorphism in the glutamate ionotropic receptor kainate-1 (*Grik1*) gene of a genetically audiogenic seizure model

**DOI:** 10.3389/fnmol.2023.1322750

**Published:** 2024-01-05

**Authors:** Sandra M. Díaz-Rodríguez, M. Javier Herrero-Turrión, Carlos García-Peral, Ricardo Gómez-Nieto

**Affiliations:** ^1^Institute of Neuroscience of Castilla y León (INCYL), University of Salamanca, Salamanca, Spain; ^2^Institute for Biomedical Research of Salamanca (IBSAL), Salamanca, Spain; ^3^Department of Cell Biology and Pathology, Faculty of Medicine, University of Salamanca, Salamanca, Spain; ^4^Neurological Tissue Bank INCYL (BTN-INCYL), Salamanca, Spain

**Keywords:** animal models of epilepsy, GASH/Sal, GluK1, glutamatergic system, kainate receptor, neurotransmission, seizure susceptibility, synaptic dysfunction

## Abstract

Genetic abnormalities affecting glutamate receptors are central to excitatory overload-driven neuronal mechanisms that culminate in seizures, making them pivotal targets in epilepsy research. Increasingly used to advance this field, the genetically audiogenic seizure hamster from Salamanca (GASH/Sal) exhibits generalized seizures triggered by high-intensity acoustic stimulation and harbors significant genetic variants recently identified through whole-exome sequencing. Here, we addressed the influence of the missense single-nucleotide polymorphism (C9586732T, p.His289Tyr) in the glutamate receptor ionotropic kainate-1 (*Grik1*) gene and its implications for the GASH/Sal seizure susceptibility. Using a protein 3D structure prediction, we showed a potential effect of this sequence variation, located in the amino-terminal domain, on the stability and/or conformation of the kainate receptor subunit-1 protein (GluK1). We further employed a multi-technique approach, encompassing gene expression analysis (RT-qPCR), Western blotting, and immunohistochemistry in bright-field and confocal fluorescence microscopy, to investigate critical seizure-associated brain regions in GASH/Sal animals under seizure-free conditions compared to matched wild-type controls. We detected disruptions in the transcriptional profile of the *Grik1* gene within the audiogenic seizure-associated neuronal network. Alterations in GluK1 protein levels were also observed in various brain structures, accompanied by an unexpected lower molecular weight band in the inferior and superior colliculi. This correlated with substantial disparities in GluK1-immunolabeling distribution across multiple brain regions, including the cerebellum, hippocampus, subdivisions of the inferior and superior colliculi, and the prefrontal cortex. Notably, the diffuse immunolabeling accumulated within perikarya, axonal fibers and terminals, exhibiting a prominent concentration in proximity to the cell nucleus. This suggests potential disturbances in the GluK1-trafficking mechanism, which could subsequently affect glutamate synaptic transmission. Overall, our study sheds light on the genetic underpinnings of seizures and underscores the importance of investigating the molecular mechanisms behind synaptic dysfunction in epileptic neural networks, laying a crucial foundation for future research and therapeutic strategies targeting GluK1-containing kainate receptors.

## 1 Introduction

Normal brain functioning requires a complex molecular interplay to mediate the proper neuronal signaling between many distinct structures. This implies an orchestrated regulation and interaction of multiple intracellular signaling and gene expression pathways that dictate the overall neuronal responses. However, the normal neuronal signaling becomes aberrant in epilepsy that is characterized by elevated neuronal hyperactivity, enhanced synchrony, and altered neurotransmitter balance in restricted regions of the brain that might eventually spread throughout many other areas. Switch in molecular and cellular mechanisms leads to ictogenesis and epileptogenesis in those brain regions to finally drive a myriad of epilepsy symptoms, including seizures as the most notable behavioral manifestation of such neuronal network overactivity (Fisher et al., 2005; Pitkänen and Engel, 2014). The relationship between the glutamatergic system and epilepsy has stimulated broad public concern, and hence studies focusing on the role of glutamate receptors in epilepsy as well as the contribution of animal models to the understanding of epileptogenesis are of the greatest interest (reviewed in Wang et al., 2022). Glutamate, the predominant excitatory neurotransmitter in the mammalian central nervous system, acts on a variety of receptor proteins that are classified in NMDA (*N*-methyl-D-aspartate), AMPA (α-amino-3-hydroxyl-5-methyl-4-isoxazole-propionate), and kainate receptors based on the agonist that activates them (reviewed in Meldrum, 2000). AMPA and NMDA receptors serve as primary mediators of excitatory neurotransmission in the brain (Traynelis et al., 2010), playing a crucial role in both the initiation and spread of seizures (reviewed in Casillas-Espinosa et al., 2012). Kainate receptors are formed by tetrameric combinations of several subunits, namely GluK1, GluK2, GluK3, GluK4, and GluK5, formerly referred to as GluR5, GluR6, GluR7, KA1, and KA2, respectively. Of these, GluK1-3 may form functional homomeric or heteromeric receptors that included many and varied structural isoforms arising from the alternative splicing of the corresponding pre-mRNAs (Pinheiro and Mulle, 2006; Lerma and Marques, 2013). Kainate receptors are widely distributed pre- and postsynaptically on different cell types in the brain, taking part in the control of synaptic networking, regulation of neurotransmitter release, modulation of both excitatory and inhibitory transmission, and in enhancement of neuronal excitability (Contractor et al., 2011).

**FIGURE 1:**
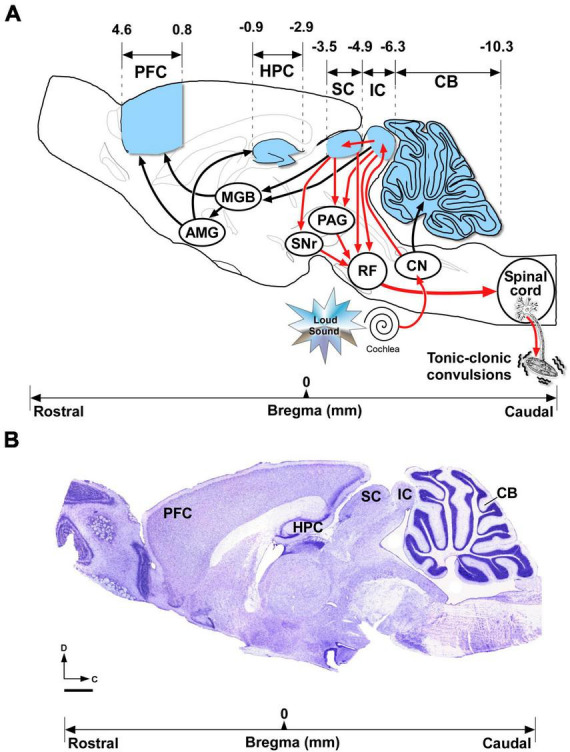
Brain areas included in the gene expression, immunoblotting and immunohistochemical analyses. **(A)** Overview of the brain structures that might be involved in the neuronal network associated with acute and chronic audiogenic seizures in genetically models of audiogenic seizures. The seizure-prone neuronal network (depicted with red arrows) involves alterations of bottom-up auditory inputs from the cochlea receptor, throughout the brainstem auditory pathway (cochlear nucleus) to the inferior colliculus (IC) (Faingold et al., 1992; Ribak and Morin, 1995; Sánchez-Benito et al., 2020). The aberrant neuronal activity in the IC initiates the audiogenic seizure that spreads throughout multiple projections targeting the superior colliculus to the substantia nigra, the periaqueductal gray, and the brainstem reticular formation, which in turn projects to the spinal cord, ultimately mediating the tonic-clonic convulsions (Garcia-Cairasco, 2002). During frequent repetitive audiogenic seizures, the seizure network might extend toward other brain regions (projections depicted with black arrows) such as the medial geniculate body, the amygdala, hippocampus, and prefrontal cortex (Faingold, 2012). The cerebellum that receives auditory inputs might be involved in the audiogenic seizure network (Mennink et al., 2020; Streng and Krook-Magnuson, 2021). The brain regions depicted in blue were selected for gene expression, immunoblotting and immunohistochemical analyses in the GASH/Sal model. **(B)** Representative Nissl-stained section of the sagittal section of the hamster brain, in which the sketch shown in **(A)** was based, shows the brain areas included in the present study. Bregma reference coordinates (in mm) were used for selection of those brain regions. Scale bar = 1 mm. AMG, amygdala; MGB, medial geniculate body; CB, cerebellum; CN, cochlear nucleus; PAG, periaqueductal gray; IC, inferior colliculus; HPC, hippocampus; PFC, prefrontal cortex; RF; reticular formation; SC, superior colliculus; SNr, substantia nigra (Image shown in (**B**) was adapted from the hamster brain atlas of Morin and Wood (2001), and the corresponding bregma data was obtained from the same source).

There is clear evidence to strongly support that kainate receptors might be involved in the excitatory to inhibitory imbalances linked to epilepsy (reviewed in Lerma and Marques, 2013). Indeed, kainate is widely used in rodents to induce acute brain seizures and, after repetitive kainic injections, as a chronic model of temporal lobe epilepsy (reviewed in Ben-Ari, 2012). The proconvulsant actions of kainic acid are largely mediated by the activation of kainate receptors, in which their subunit composition and their possible action mechanisms has been widely studied and hotly debated (reviewed in Falcón-Moya et al., 2018). Thus, several studies suggested that the glutamate ionotropic receptor kainate type subunit 1 (GluK1) has a principal role in the activation of kainate receptors that favor the imbalance between excitation and inhibition in kainate-induced seizures (Rodríguez-Moreno et al., 1997; Fritsch et al., 2014; Falcón-Moya et al., 2018). Some studies suggest that kainate receptors containing the GluK1 subunit represent promising candidates for seizure prevention (Khalilov et al., 2002; Smolders et al., 2002; Rogawski et al., 2003), while others failed to confirm the pharmacological inhibition of GluK1-kainate receptors as an epilepsy treatment strategy (Fritsch et al., 2014). The uncontested fact is that bench to bedside ventures in epilepsy research cannot be accomplished without the contribution of preclinical animal models of seizures and epilepsy syndromes. Besides the experimental models induced by chemical means (e.g., the aforementioned model of kainate-induced seizures), the genetically audiogenic seizures models offers the following advantages: (1) The seizure susceptibility is inherited and there are no need of using exogenous chemicals or any experimental procedure to become susceptible, avoiding thus incompatibilities with experimental designs; (2) Seizures are induced on demand by the investigator when the animal is exposed to intense acoustic stimulation; (3) The overwhelming detailed information at the molecular, cellular and behavioral levels available for the research community that makes genetically audiogenic seizures models ideal to elucidate mechanisms and neuronal substrates underlying the seizure genesis and propagation (Ross and Coleman, 2000; Kandratavicius et al., 2014). Among the variety of genetically seizure-prone strains of rodents, the only scientifically available hamster that exhibits seizure susceptibility to sound is the genetic audiogenic seizure hamster from Salamanca (GASH/Sal) that presents an autosomal recessive inheritance pattern (Muñoz et al., 2017). In the GASH/Sal strain, high-intensity acoustic stimulation causes generalized tonic-clonic seizures that can be reduced or eliminated after administration of anticonvulsant compounds (Barrera-Bailón et al., 2013, 2017; Werner and Coveñas, 2017; Cabral-Pereira et al., 2021). There is currently a wide range of comprehensive knowledge regarding the neuroethological (Barrera-Bailón et al., 2013, 2017), electrophysiological (Carballosa-Gonzalez et al., 2013), neurochemical (Prieto-Martín et al., 2017; Fuerte-Hortigón et al., 2021), molecular (López-López et al., 2017; Díaz-Casado et al., 2020; Díaz-Rodríguez et al., 2020; Bonet-Fernández et al., 2023) and morphological (Sánchez-Benito et al., 2017, 2020) features underlying the audiogenic seizures in the GASH/Sal model. At the molecular genetic level, a study using whole-exome sequencing in GASH/Sal and wild-type hamsters identified 3 high-impact and 15 moderate-impact genetic variants (Díaz-Casado et al., 2020). Among these missense single-nucleotide variants, it is noteworthy the substitution of C by T at position 9586732 of the *Grik1* gene that encodes the GluK1 protein, in which the His residue at position 289 is replaced for a Tyr (p.H289Y) (Díaz-Casado et al., 2020). This finding is extremely interesting since genetic variants of *Grik1* has been linked to epilepsy genetic risk in humans (Genome-wide association studies by the International League Against Epilepsy Consortium on Complex Epilepsies, 2018). Thus, *Grik1* polymorphisms confers susceptibility to juvenile absence epilepsy (Sander et al., 1997), and variations in the non-coding region of this gene, near regulatory sequences, could alter gene expression without affecting receptor structure (Izzi et al., 2002). Here, we explore for the first time the effects of the missense single-nucleotide polymorphism (C9586732T, p.His289Tyr) in the *Grik1* gene of the GASH/Sal model using an *in silico* 3D modeling protein structure. The brain structures that contribute to the seizure susceptibility, genesis, and propagation as well as those nuclei that might be recruited after repeated acoustic stimulation has been widely studied, coming out very similar among the different audiogenic seizure models (summarized in Figure 1). Activation of auditory pathways are required for the onset and progression of seizures in all audiogenic seizure models, and many studies pointed out that glutamate and glutamate receptors in the inferior colliculus (IC), a critical integration center in the auditory midbrain pathway, are essential to initiate, audiogenic seizures (Faingold, 2012; Faingold et al., 1992; Ross and Coleman, 2000; Garcia-Cairasco, 2002). In the GASH/Sal, the alterations at the morphological and molecular connectome level of the primary acoustic pathway form part of the GASH/Sal seizure-prone neural network that includes aberrant glutamatergic neurotransmission in the flow of sound processing from the inner ear to the cochlear nucleus to the IC (Sánchez-Benito et al., 2017, 2020). As a result, the IC is embedded in a web of pathologic connections that spread aberrant neuronal activity through multiple brain areas to finally drive the tonic-clonic convulsions (Sánchez-Benito et al., 2020). Despite these substantial advances to elucidate the glutamatergic system in genetic audiogenic seizure models (Ross and Coleman, 2000), the role of kainate receptors in the audiogenic seizure network is still waiting to be determined. As a novelty in the present article, we studied the *Grik1* gene expression as well as the protein levels of GluK1 in the GASH/Sal seizure network (Figure 1), using consolidated methodologies and tools available for the characterization of audiogenic seizures models (Bosque et al., 2021). The absence of specific antibodies against different kainate receptor subunits has limited the study of the receptor distribution in brain for a long time, however, new antisera are now available, and it is known that GluK1 is present in hippocampal and cortical interneurons (Lerma and Marques, 2013). The distribution of GluK1 in the audiogenic seizure network of genetic seizure-prone rodent models remains unknow and it has never been explored in the GASH/Sal strain. In the present study, we further conducted immunohistochemical analysis at bright-field and confocal scanning microscopy to examine the distribution of GluK1 in critical brain structures involved in the seizure neuronal network of the GASH/Sal (Figure 1). Since the outcomes of these experiments were based on the comparisons with age-matched wild-type Syrian hamsters, our study also provides valuable information of the GluK1 protein distribution in the brain of the *Mesocricetus auratus*. The overall goal of this study was to shed light on the effects of genetic alterations in a model organism with inherited propensity for developing seizures as well as delve into the knowledge of kainate receptors in the seizure-associated neural networks.

## 2 Materials and methods

### 2.1 *In silico* based protein 3D structure analysis and modeling

An *in silico* approach was used to analyze the impact of a missense single-nucleotide polymorphism in the *Grik1* gene of the GASH/Sal model. This computational method involved the utilization of diverse bioinformatics software tools for a comprehensive analysis of genetic variations within the context of protein 3D structures. Using the AlphaFold2 deep learning-based protein structure prediction system (Jumper et al., 2021), we investigated the impact of the specific gene variant p.H289Y on the structure of the GluK1 protein in *M. auratus* (sequence XP_005073900), while exploring various aspects of protein structure and organization. AlphaFold2 uses a two-step process to predict protein structures: it trains a deep learning model on known protein structures to predict amino acid distances, and then employs a second network to generate 3D coordinates for the atoms based on these distances, resulting in a predicted 3D structure (Jumper et al., 2021). Furthermore, interatomic interactions in the wild-type and mutated residues (p.H289Y) of the GluK1 subunit were calculated using the web server Arpeggio (Jubb et al., 2017), and their lengths were determined using the molecular viewer PyMOL (The PyMOL Molecular Graphics System, Version 1.8 Schrödinger, LLC). 3D images, depicting various views of the modeled protein structures, were generated using the PyMOL system. Additionally, we employed the Cortona Movie Maker software (Cortona; Parallel Graphics, Boston, MA, USA) to create video recordings at a resolution of 1024 × 768 pixels using the VRLM files of the corresponding 3D renderings. To assess the effect of the missense mutation on the thermodynamic stability of the protein (ΔΔG*^Stability^*), several protein stability predictors were employed: Dynamut2, INPS3D, FoldX, and MAESTRO. These computational stability predictors functioned as web interfaces that evaluated changes in protein folding or interaction energies due to mutations (expressed as ΔΔG, representing the change in Gibbs free energy). DynaMut2 incorporated dynamics into the mutation analysis, enabling a precise evaluation of the mutation’s impact on protein stability (Rodrigues et al., 2021). INSP3D considered sequence and physicochemical properties, as well as structure-derived features such as solvent accessibility and local energy differences (Savojardo et al., 2016). FoldX utilized an empirical force field to rapidly assess the effects of mutations on the stability, folding, and dynamics of proteins and nucleic acids (Schymkowitz et al., 2005). Lastly, MAESTRO employed statistical scoring functions and various machine learning approaches to generate multi-agent predictions (Laimer et al., 2016). For the Dynamut2 and INPS3D predictors, the mutation was considered stabilizing if the ΔΔG value was greater than 0 and destabilizing if it was less than 0, whereas for the FoldX and MAESTRO predictors, the mutation was considered stabilizing if the ΔΔG value was less than 0 and destabilizing if it was greater than 0.

### 2.2 Animal experiments and ethical statement

A total of 16 Syrian golden hamsters (*M. auratus*) were used in this study, specifically 8 wild-type hamsters (RjHan:AURA) from Janvier Labs (Le Genest-Saint-Isle, France) and 8 GASH/Sal animals from the inbred strain maintained at the vivarium of the University of Salamanca (USAL, Spain). The experimental design of the current study was based on comparisons with wild-type hamsters that were used as a control group. All control and GASH/Sal animals matched age (4 months old), gender (male), housing, handling, and care, in order that these variables can be ruled out as alternative explanations of any observed differences. The age of 4 months was selected as it was previously reported that the GASH/Sal strain exhibits the maximum susceptibility to seizures from 2 to 4 months of age (Muñoz et al., 2017). All GASH/Sal animals were naïve to sound-induced seizures, and hence, were not receiving any high-intensity sound stimulation to trigger audiogenic seizures. All procedures and experimental protocols were performed in accordance with the guidelines of the European Communities Council Directive (2010/63/UE) for the care and use of laboratory animals and approved by the Bioethics Committee of the University of Salamanca (approval number 375). All efforts were made to minimize the number of animals and their suffering. The animals were maintained in Eurostandard Type III cages (Tecniplast, Italy), with Lignocel bedding (Rettenmaier Iberica), 14/10 light/dark cycle, 22–24°C room temperature (RT) with *ad libitum* access to food (Tecklad Global 2918 irradiated diet) and water. Communities of 2–3 animals were housed in groups until the beginning of the study. A total of 10 animals (5 controls and 5 GASH/Sal) were processed for gene expression analysis of *Grik1* as well as immunoblotting to compare the levels of the corresponding encoded protein GluK1. The brain tissue from these 10 animals was obtained by inducing general anesthesia using 4% isoflurane (Forane, Abbott, IL, USA) vaporized in 100% oxygen at a flow rate of 1 L/min, followed by rapid decapitation for euthanasia. Subsequently, their brains were rapidly removed, hemisected, and frozen until use in downstream analysis (half of the brain for mRNA expression analysis and the other half for immunoblotting). In separate set of experiments, 6 animals (3 controls and 3 GASH/Sal) were processed for immunohistochemistry to determine the distribution of GluK1 in the brain tissue, following the procedure outlined below. In all animal experiments, the target study area included the following specific brain regions: cerebellum, inferior and superior colliculi, hippocampus, and prefrontal cortex (Figure 1). As indicated in the introduction, the selection of these specific brain areas was made based on their potential role in the seizure-associated neuronal network, in which the mRNA and protein expression patterns as well as the immunostaining may differ between wild-type and GASH/Sal animals. In each set of experiments, tissue samples were obtained and processed in parallel for both animal groups as previously done by our research team (Gómez-Nieto et al., 2014a; Sánchez-Benito et al., 2017, 2020). Hence, the disparities observed were unrelated to the passage of time or any other experimental variables, including incubation periods, temperature, or sample manipulations.

### 2.3 Real-time quantitative reverse transcription PCR (RT-qPCR)

Total RNA was sequentially extracted using 30–70 mg of brain tissue and purified using a commercial kit (ReliaPrep™ RNA Tissue Miniprep, Z6112). The RNA was quantified, and its quality assessed by using an Agilent 2100 Bioanalyzer. Only samples with an RNA integrity number (RIN) >8.0 were used. The quantitative reverse transcription real-time polymerase chain reaction (RT-qPCR) procedure was carried out following the protocol routinely used by our research group (Díaz-Rodríguez et al., 2020; Sánchez-Benito et al., 2020; Cabral-Pereira et al., 2021). Briefly, complementary DNA (cDNA) was synthesized using messenger RNA (mRNA) contained in purified RNAs via a retrotranscription enzymatic reaction using the kit ImProm-II™ Reverse Transcription System (Promega). Total RNA (2 μg) was mixed with oligo-dT and random hexamer primers for reverse-transcription into cDNA at 37°C for 2 h into a 20 μL volume and stored at −20°C of temperature until use. In all cases, a reverse transcriptase negative control was used to test genomic DNA contamination. Subsequently, qPCR was conducted using the SYBR Green method with a 2 × Master Mix (#4367659, Applied Biosystems) as previously described (Herrero-Turrión et al., 2014). In brief, each reaction consisted of 10 μL of Master Mix, 0.4 μL of each primer (Table 1), 3 μL of the respective cDNA sample (ranging from 10 to 100 ng depending on the primer pair), in a different serial cDNA quantity for each gene, and MilliQ water (RNA-free) to reach a final volume of 20 μL. The amplification reaction was performed in the QuantStudio™ 7 Flex Real-Time PCR System (Applied Biosystems) under the following conditions: 10 min at 95°C followed by 40 cycles of 15 s at 95°C, and 30 s at 60°C depending on each pair of primers. RT-qPCR experiments were performed in replicates of 5 samples and conducted in triplicate. The comparative cycle threshold (Ct) method was used for presenting quantitative data (Schmittgen and Livak, 2008). Following removal of outliers (Burns et al., 2005), raw fluorescence data were used to determine PCR amplification efficiency (E) according to the equation *E* = [10 ^[–1/*slope*]^–1] × 100. All amplifications had an *E*-value of 100 ± 10%, with an *E*-value close to 100% serving as an indicator of effective amplification. Before quantification of amplified DNA samples, E analysis for each primer (specific for each targeted gene) was performed (Table 1). These analyses allowed us to establish the expression dynamic range for each primer. To decide the most stable reference gene for RT-qPCR data normalization, two candidates [β-actin (*Actb*) and tubulin (*Tbp*)] were selected and their expression was measured by NormFinder software (Andersen et al., 2004) that calculates intra- and intergroup variations in gene expression. Our study identified *Actb* as the most suitable reference gene, and hence, the mean Ct value and primer *E*-value of *Actb* were used for data normalization. The relative gene expression value of each transcript was calculated following the comparative 2^–ΔΔ*Ct*^ method as used previously (Damasceno et al., 2020; Sánchez-Benito et al., 2020). Finally, a negative template-free (water) control reaction was used in all RT-qPCRs and the control group was used as the calibration sample.

**TABLE 1:** List of oligonucleotide primers used for RT-qPCRs.

Gene target	ID transcript Ensembl *Mesocricetus auratus*^a^	Primer forward	Primer reverse	Size of products (bp)	E^b^ (%)
*Grik1*	*ENSMAUG00000000865*	TGTTCGCTTTAGATCTGGAACTC	TCATGCCATCAAGAAGACCA	165	93
*Actb*	*ENSMAUG00000008763*	AGCCATGTACGTAGCCATCC	ACCCTCATAGATGGGCACAG	105	97
*Tbp*	*ENSMAUG00000019343*	TGTATCCACGGTGAATCTTGG	GAAAATCAGCGCAGTTGTCC	139	95

“^a^”Indicates the identifier (ID) of each gene in the corresponding Ensembl sequences of the Syrian hamster (*M. auratus*).

“^b^”Indicates percentage of qPCR primer efficiency (E).

### 2.4 Primary antibody against GluK1

In this study, we used immunoblotting to assess GluK1 protein levels and immunohistochemistry to visualize its distribution. The polyclonal anti-GluK1 antibody (catalog No. ab118891; Abcam) was generated in rabbits against a synthetic peptide corresponding to the central region of the human GluK1 within 380–430 amino acids, and the reactivity was validated in human and rat, showing the highest tissue specificity in the cerebellum. In Western blot analysis, the anti-GluK1 antibody recognizes a single band migrating at approximately 104 kDa (as per the manufacturer’s technical information). In this study, we adhered to the manufacturer’s guidelines and utilized this primary antibody for both Western blotting and immunocytochemistry. This primary antibody has been utilized successfully in prior immunohistochemical studies conducted by our research group (Díaz-Rodríguez et al., 2023) and by others (Perez-Ortiz et al., 2017). Nevertheless, as per the manufacturer’s data sheets, hamster brain tissue reactivity remains untested. To tackle this concern, as illustrated in Supplementary material 1, we initiated a multi-sequence alignment analysis aimed at assessing the variability or conservation of epitopes in hamsters. For this, we retrieved the GluK1 protein sequence from the *Grik1* gene in the NCBI protein database^1^ and conducted the analysis using the Jalview program (version 2.11.2)^2^ (Troshin et al., 2011). The multiple sequence alignment showed that the antigenic region is highly conserved for all isoforms in the hamster, thereby indicating the suitability of employing the previously mentioned Gluk1 antibody in the current study (Supplementary material 1).

### 2.5 Western blotting

Brain tissues containing each of the specific brain areas shown in Figure 1, with a weight between 30 and 75 mg, were collected from wild-type and GASH/Sal animals. Total protein extraction and isolation were carried out following homogenization of tissue samples in radioimmunoprecipitation assay (RIPA) buffer (catalog No. 9806S, Cell Signaling Technology) that contained a mixture of protease inhibitors (catalog No. 78442, Thermo Fisher Scientific Inc). Homogenates were centrifuged at ∼14,000 × *g* for 15 min to remove insoluble material. Protein concentration was determined using the Bio-Rad DC protein assay kit (catalog No. 500-0116, BIO-RAD). Next, the protein extracts were mixed with 10x NuPAGE™ Sample Reducing Agent (catalog No. NP0004, Thermo Fisher Scientific Inc) and 4x NuPAGE™ LDS Sample Buffer (catalog No. NP0007, Thermo Fisher Scientific Inc), and then boiled at 75°C for 10 min as indicated by the manufacturer’s instructions. Equal amounts of protein (30 μg) were loaded into each lane of a Bolt 10% Bis-Tris gels (catalog No. NW00100BOX, Thermo Fisher Scientific Inc) and electrophoretically separated at RT using 90 V for 35 min and subsequently 200 V for 30 min with the PowerPac™ Basic power supply (catalog No. 1645050, BIO-RAD). The separated proteins were transferred to nitrocellulose membranes using the dry immunoblotting iBlot™ 2 system for approximately 7–8 min at 20–25 V (Invitrogen/Thermo Fisher Scientific Inc). To prevent non-specific antibody binding, all membrane blots were blocked for 1 h at RT with Tris-buffered saline/Tween (TBS-T), containing 5% bovine serum albumin (BSA, catalog No. A2153-100G, Sigma Aldrich). The membranes were subsequently incubated with the rabbit polyclonal anti-GluK1 antibody (10 ug/ml) overnight for 72 h at 4°C. Then, the membranes blots were washed with TBS-T and incubated with the horseradish peroxidase-conjugated secondary antibody (goat anti-rabbit antibody, catalog No. 7074S, Cell signaling technology) at a dilution of 1:250 for 2 h at RT. The membranes were stained with SuperSignal West Pico Chemiluminescent Substrate (catalog No. 34580, Thermo Fisher Scientific Inc) and detection was performed by an enhanced chemiluminescent method which combines the MicroChemi Unit and GelCapture image acquisition software. After image capturing, β-actin protein was used as internal loading control for comparative Western blot analysis of protein signals in the samples. To analyze protein loading, the membranes were washed extensively with TBS-T before treating with Restore™ Western Blot Stripping Buffer (catalog No. 21059, Thermo Fisher Scientific Inc) for 15 min at RT. Next, the membranes were re-blocked with a 5% BSA solution in TBS-T for 1 h at RT before being incubated with a 1:10,000 diluted rabbit anti-β-actin antibody (catalog No. 4967s, Cell Signaling Technology) for 1 h at RT, and this was followed by the application of horseradish peroxidase-conjugated anti-rabbit secondary antibodies (as previously mentioned). The membranes were then treated with chemiluminescent substrate and imaged as described above. Densitometric intensities of the digitalized membranes were measured and analyzed using the ImageJ software (WS, 1997). Band densities were normalized to the corresponding loading control densities and expressed as arbitrary density units.

### 2.6 Immunohistochemistry for bright-field and confocal scanning microscopy

Brain tissue employed for immunohistochemical analysis at bright-field and confocal scanning microscopy was collected and prepared following the established procedures described in our previous reports (Sánchez-Benito et al., 2017, 2020; Fuerte-Hortigón et al., 2021). Following the administration of a lethal dose of sodium pentobarbital (60 mg/kg) and subsequent perfusion through the heart with 4% paraformaldehyde in 0.1 M phosphate-buffered saline (PBS), the brains were removed from the skull. They were then cryoprotected through immersion in a 30% sucrose solution and coronal sections were sliced at a thickness of 40 μm using a freezing sliding microtome. Promptly, the serial sections were collected in 0.1 M PBS and organized into a set of 6. Sample labeling and visualization of the GluK1 protein were carried out following the steps of the immunohistochemistry method previously used by our research group (Gómez-Nieto et al., 2008a,b). Washes were made in Tris-buffered saline (TBS) pH 7.6 and dilutions of antisera in TBS pH 7.6 containing 0.2% Triton X-100 (catalog No. T9284; Sigma). All immunostaining steps were performed at RT (∼22°C), unless stated otherwise. For brightfield microscopy analysis, free-floating sections were blocked for 1 h with 6% normal goat serum (catalog No. S-1000, Vector Labs) and then incubated with the primary antibody, rabbit anti-GluK1 (dilution 1:1000) for 72 h at 4°C. Subsequently, sections were then washed and followed incubation with the goat anti-rabbit biotinylated secondary antibody (catalog No. BA-1000, Vector Laboratories) at 1:200 dilution for 2 h. After removal of secondary antisera, the visualization of epitope-antibody interactions was developed with the avidin-biotin-peroxidase complex procedure (catalog No. PK-4000, Vectastain, Vector Labs.), and diaminobenzidine histochemistry for peroxidase without heavy-metal intensification (DAB Kit, catalog No. SK-4100, Vector Labs.). All sections were mounted on slides, dehydrated and coverslipped with Entellan^®^Neu (catalog No. 107961, Merck). Histological sections containing the specified regions of interest were examined using a light microscope (Leitz DMRB, Leica Biosystem) equipped with a digital camera (DP50, Olympus). All microscope parameters and settings for digitizing the photomicrographs remained constant across both experimental groups and for each animal. Low-magnification images were taken with the 4x or 10x objective lens, and high magnification images were taken with a 40x or 100x objective lens (oil immersion). Morphometric measurements (diameter and area) of labeled structures were achieved analyzing high magnification images with the ImageJ software (version 1.53c) as described in our previous report (Gómez-Nieto et al., 2014b). For immunofluorescence analysis, non-specific binding sites were blocked for 1 h with 6% normal goat serum (catalog No. S-1000, Vector Labs) and then the sections followed incubation with a mixture of two primary antibodies, rabbit polyclonal anti-GluK1 (dilution 1:1000) and mouse monoclonal anti-NeuN (dilution 1:500, catalog No. MAB377, Millipore Sigma) for 72 h at 4°C. Thereafter, the sections were rinsed extensively and reacted for 2 h with the secondary antibodies using the VectaFluor™ Duet Immunofluorescence Double Labeling Kit [#DK-8818, DyLight^®^ 488 Anti-Rabbit IgG (green)/DyLight^®^ 594 Anti-Mouse IgG (red), Vector Labs]. Finally, sections were mounted on slides and coverslipped with VECTASHIELD^®^ mounting medium for preserving fluorescence, containing the DAPI counterstain (4′,6-diamidino-2-phenylindole; catalog No. H-1200; Vector Labs.). The fluorescent DAPI dye, which labels the nuclear DNA of cells, and the NeuN-immunolabeling, which is specific to a nuclear protein expressed by nearly all neurons (with few exceptions like Purkinje Neurons as noted by Weyer and Schilling, 2003), were used to accurately delineate the cytoarchitecture of the brain nuclei. The sections processed for immunofluorescence were studied on a Leica Stellaris 8 confocal laser coupled to a Leica DMi8 inverted microscope, using the appropriate settings for detection of DyLight^®^ 594 (red), DyLight^®^ 488 (green) and DAPI fluorochromes. Representative images for illustration were collected as stacks with a z-step of 0.3 μm slices, using a 63x/NA1.4 oil-immersion objective lens and different digital zoom levels. The maximum intensity and stereo Z-projections of fluorescence confocal microscopy images (10–12 μm thickness) were used as methods of 3D visualization. We obtained images and animated-video documents from the 3D renderings using the image and movie editor in the Leica Application Suite X (LAS X software, version 4.2.0), and we completed the final composition of the videos using the Camtasia Studio software (version 8.3.0). In all immunohistochemical experiments, the absence of staining in the preparations was observed when the primary antibody was omitted. For visualization of the results, the stereotaxic atlas of the golden hamster brain (Morin and Wood, 2001) was used as an anatomical reference, in which histological sections were caudo-rostrally arranged. The photomicrographs were processed with minor modifications in contrast using Adobe Photoshop CS2 and the final composition of the figures was achieved with the Canvas 14 software.

### 2.7 Statistical analysis

Statistical analyses including relative values of gene expression and western blot-band intensities were performed using the SPSS-IBM software, version 20 (SPSS Inc., Chicago, IL, USA). For each statistical analysis, comparisons between control and GASH/Sal animals were performed with analysis of variance (*post-hoc* analysis with Fisher’s test) and Student’s *t*-test. The differences were considered statistically significant with a *p*-value < 0.05 (*), *p*-value < 0.01 (**) and *p*-value < 0.001 (***). All the quantitative data were presented as mean value ± standard error of the mean (SEM) and were plotted using GraphPad Prism (version 8). The GraphPad Prism software was further used to automatically create heatmaps, in which the squares were color coded according to the mean values of relative mRNA expression (RT-qPCR) or normalized densitometry (Western blot) for each brain structure, relative to the values obtained for the IC.

## 3 Results

### 3.1 Effect of the single-nucleotide polymorphism in the 3D protein structure

**FIGURE 2:**
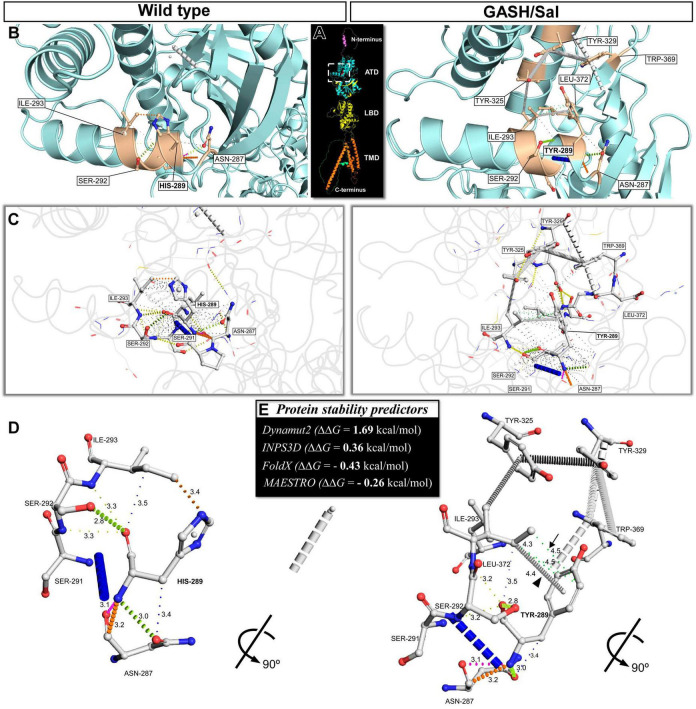
The missense single-nucleotide polymorphism p.H289Y provides new hydrophobic interactions that lead to improved GluK1 protein stability. **(A)** Ribbon representation of the predicted monomeric form of the GluK1 receptor illustrates its domain organization. These domains, namely the amino-terminal domain (ATD), ligand-binding domain (LBD), and transmembrane domain (TMD), are highlighted in different colors. **(B)** Close-up views corresponding to the white dashed box in **(A)** showcase the residual local environment in the ATD, in which the substitution of histidine-289 to tyrosine was found. The left panel shows the wild-type protein structure, and the right panel shows the mutated form in the *M. auratus* (structures predicted by AlphaFold). The highlighted residues with side-chain atoms shown with sticks are the mutated site and the neighboring residues. **(C)** Roving detail corresponding to the white dashed box in **(A)** features the molecular interactions between the mutated site and the neighboring residues. These interactions are represented by colored dashed lines, with thicker dashes indicating overlapping van der Waals’ radii and the thinnest dashes representing “proximal” interactions beyond van der Waals’ radius overlap but within 5 Å. **(D)** Protein backbone representations showcasing a 90° rotation of the 3D depictions shown in **(B,C)**. This 3D representation was simplified by hiding the undefined-proximal interactions to provide a clear visualization of the formation of new hydrophobic-proximal interactions (green dashed lines, arrow) and atom ring interactions (carbon–π, arrowhead) in the mutated structure, in comparison to the wild-type. The distances between atoms involved in each interaction are displayed in Å units. **(E)** Prediction of the change in fold stability (ΔΔG in kcal/mol) of the GluK1 protein due to the substitution of histidine-289 to tyrosine. ΔΔG values ≥0 were considered as stabilizing and ΔΔG values < 0 were destabilizing for Dynamut2 and INPS3D predictors, while ΔΔG values ≤0 were considered as stabilizing and ΔΔG values >0 were destabilizing for FoldX and MAESTRO predictors. Notice that all predictors consistently yielded stabilizing results.

Our first approach to provide molecular insights into the potential consequences of the missense single-nucleotide polymorphism (C9586732T) in the *Grik1* gene of the GASH/Sal hamster was to generate a predicted 3D model of the corresponding protein structure. This single-nucleotide variant replaces in the amino acid sequence a histidine by a tyrosine at the codon 289 (p.H289Y, numbering according to the isoform X2; Uniprot A0A1U7QK31) in the GluK1 protein. The histidine residue at position 289 is in the amino-terminal domain (ATD) that together with the ligand binding domain form the extracellular moiety of the GluK1 protein (Supplementary material 1). To assess the potential impact of the p.H289Y polymorphism, we employed the protein structure prediction tool AlphaFold2, and subsequently analyzed the structural environment of the mutated residue. Figure 2A provides a visual representation of the ribbon structure, presenting the predicted monomeric form of the GluK1 receptor. Additionally, Supplementary material 2, which includes a video featuring dynamic rotations, offers a comprehensive and detailed visual depiction, highlighting the distinct domain organization of the receptor. Upon comparing the wild-type and mutated protein structures, we observed a consequential modification in the local environment of the regulatory region R2, located in the lower lobe of the ATD, which adopts a clamshell-like structure (Figures 2B, C and Supplementary material 2). Notably, our analysis revealed significant disparities between the wild-type and mutated structures, particularly in the emergence of new hydrophobic-proximal interactions and carbon-π interactions (atom ring interactions) in the mutated structure (Figure 2D; see video in Supplementary material 2). We further employed several computational stability predictors, including Dynamut2, INPS3D, FoldX, and MAESTRO, to assess the predicted change in fold stability (ΔΔG in kcal/mol). Our analysis consistently showed a favorable trend toward stabilization, as depicted in Figure 2E. Dynamut2 and INPS3D categorized ΔΔG values ≥0 as stabilizing, while FoldX and MAESTRO considered ΔΔG values ≤0 as indicative of stabilization. The specific predicted values for ΔΔG^Stability^ were as follows: 1.60 kcal/mol for Dynamut2, 0.36 kcal/mol for INPS3D, −0.43 kcal/mol for FoldX, and −0.26 kcal/mol for MAESTRO (Figure 2E). Notably, consistent results emerged across all stability predictors, indicating that the p.H289Y polymorphism presumably led to protein stabilization. Given the high percent identity (>97%, Supplementary material 1D) observed in the amino acid sequence of the GluK1 protein across various species such as mice, rats, and humans, it can be inferred that the presumed impact of this point mutation would be similar if it occurred in any of these species. Together, these results suggested that the single-point mutation p.H289Y leads to protein stabilization by increasing intermolecular interactions, as compared with the wild-type GluK1 receptor.

### 3.2 Gene expression of *Grik1*

**FIGURE 3:**
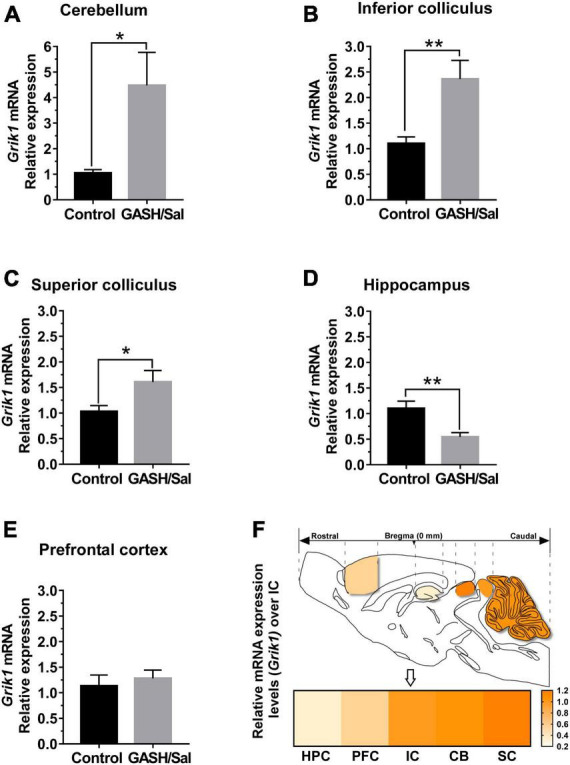
mRNA expression levels of *Grik1* gene within the seizure neural network of the GASH/Sal. Histograms show relative quantities of transcripts of the *Grik1* gene in the cerebellum [CB, **(A)**], the inferior colliculus [IC, **(B)**], the superior colliculus [SC **(C)**], the hippocampus [HPC, **(D)**], and the prefrontal cortex [PFC, **(E)**] of GASH/Sal animals. Notice that disruption of mRNA expression levels was found in the CB, the IC, the SC, and the HPC, whereas no differential gene expression was found in the PFC. Each bar in the histograms is an average ± S.E.M. Asterisks indicate statistical significance of gene expression in the brain regions of the GASH/Sal as compared to the control animals (*p-value < 0.05; **p-value < 0.01). **(F)** Sketch of the sagittal section of the GASH/Sal brain shows differences in relative transcript abundance between the structures of the seizure neuronal network. The color in the heat map represents the relative difference in mRNA expression levels of the *Grik1* gene between the control and GASH/Sal hamsters in each brain structure as compared to the IC (arrow), which were set equal to 1.0 in the heat map. For clarity the brain structures are ordered by the increasing color intensity in the lower part of the panel.

In a subsequent experimental approach, we quantified the transcriptional abundance of the *Grik1* gene in pivotal brain nuclei associated with the neuronal network involved in audiogenic seizures (Figure 3). Thus, brain tissue containing the specific brain areas shown in Figure 1 was freshly dissected from control and GASH/Sal animals. Quantitative gene expression data were normalized using *Actb* (β-actin) as internal reference gene. There were no significant differences in the number of cycles to reach the amplification threshold for *Actb* in both animal groups, indicating that the sample preparation was consistent. Raw gene expression data can be found in Supplementary material 3. Compared to controls, the mRNA expression levels of the *Grik1* gene in the GASH/Sal was significantly higher in the cerebellum (*p*-value < 0.05; Figure 3A) as well as in the inferior and superior colliculi (*p*-value < 0.01 and *p*-value < 0.05, respectively; Figures 3B, C). On the other hand, comparison of gene expression/*Actb* ratios showed that expression of *Grik1* was significantly lower in the GASH/Sal hippocampus (*p*-value < 0.01; Figure 3D). In the prefrontal cortex, the gene expression of *Grik1* was not significantly different between control and GASH/Sal animals (Figure 3E). To determine the differences in relative transcript abundance between the structures of the seizure-associated neuronal network, we compared the results of the gene expression analysis in each structure with those obtained in the epileptogenic focus. The heatmap showed that the relative difference in mRNA expression levels of the *Grik1* gene was higher in the cerebellum and the superior colliculus, being this last the one with the highest increased differences as compared to the IC (Figure 3F). On the contrary, the brain structures exhibiting lower relative differences in mRNA expression levels than the IC were the prefrontal cortex, and in particular the hippocampus, that decreased notably over the epileptogenic focus (Figure 3F). In sum, these results indicated a disruption in the transcriptional profile of *Grik1* gene in the GASH/Sal model with differences in gene expression between the structures involved in the seizure neuronal network.

### 3.3 GluK1 protein levels

**FIGURE 4:**
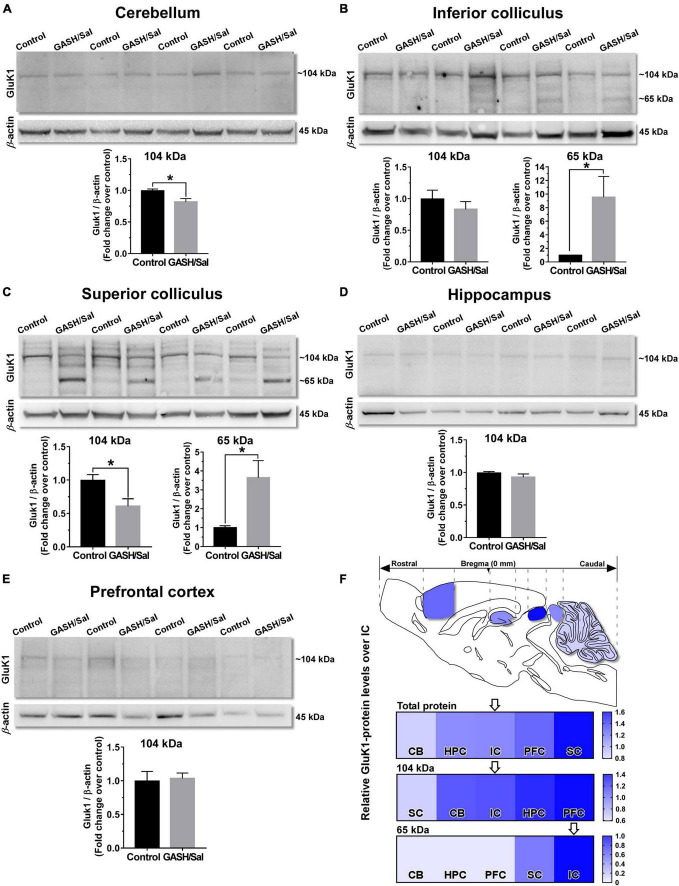
GluK1-protein levels within the seizure neural network of the GASH/Sal. Western blot analysis of relative GluK1-protein levels between the control and GASH/Sal hamsters in the cerebellum [CB, **(A)**], inferior colliculus [IC, **(B)**], superior colliculus [SC, **(C)**], hippocampus [HPC, **(D)**], and prefrontal cortex [PFC, **(E)**]. Representative western blot images of protein bands for the GluK1 receptor in each of the brain areas are depicted in the upper part of each panel. Histograms showing a quantitative analysis of GluK1-protein levels are depicted in the lower part of each panel. Each bar graph represents the mean ± standard deviation of the western blot-band intensities that were compared to wild-type values (control animals), which were set equal to 1.0. Notice that a band of the predicted molecular weight (∼104 kDA) for GluK1 was detected in all the analyzed brain areas, and in addition a second band of ∼65 kDA was observed in the IC and SC of the GASH/Sal. Note that protein levels of GluK1 was significantly different between control and GASH/Sal animals in the CB, the IC, and the SC. *p-value ≤ 0.05. **(F)** Sketch of the sagittal section of the GASH/Sal brain shows differences of total GluK1-protein levels between the structures of the seizure neuronal network. The color in the heat map represents the relative GluK1-protein levels between the control and GASH/Sal hamsters in each brain structure as compared to the IC (arrow), which were set equal to 1.0 in the heat map. Additional heat maps for the two detected GluK1 isoforms (∼104 and ∼65 kDa) are shown in the lower part of the panel. For clarity the brain structures are ordered by the increasing color intensity in all heat maps.

To further study the protein expression levels of the GluK1 receptor, we next detected the GluK1 protein levels by western blotting in the key brain structures (depicted in Figure 1) of the wild-type and GASH/Sal animals. The protein levels of GluK1 were normalized by calculating the intensity ratio of the bands according to the corresponding levels of the β-actin protein. As shown in Figure 4, compared with the controls, the protein content varied between the different brain regions, showing the band with the expected size (∼104 kDa) in all the analyzed structures and, additionally, a second band of ∼65 kDa in the inferior and superior colliculi of the GASH/Sal. This is in stark contrast with the control animals, in which the band of ∼65 kDa was faint or absent in all brain structures (Figure 4). In the cerebellum, the analysis showed that the GluK1 proteins levels significantly decreased compared with the control group (*p*-value < 0.05; Figure 4A). The band of ∼104 kDa showed no significant difference between control and GASH/Sal animals in the IC, whereas the 65-kDa band was remarkable detected in the GASH/Sal (Figure 4B). Bands of ∼104 and ∼65 kDa were also detected in the superior colliculus, showing a significant decrease and increase compared with the control group, respectively (*p*-value < 0.05; Figure 4C). In the hippocampus and the prefrontal cortex, there were no significant differences in the protein levels of GluK1 and the 65-kDa band had no signal (Figures 4D, E). To determine the differences in relative GluK1 levels between the structures of the seizure-associated neuronal network, we compared the normalized signal intensities of the GluK1 bands in each structure with those obtained in the epileptogenic focus. The heatmap showed that the total protein levels of GluK1 were higher in the prefrontal cortex and the superior colliculus, being this last structure with the highest increased differences as compared to the IC (Figure 4F). On the contrary, the brain structures exhibiting lower GluK1 levels than the IC were the hippocampus and the cerebellum (Figure 4F). Taken separately, the relative protein levels of the 104-kDa band were higher in the hippocampus and, particularly to a greater extend, the prefrontal cortex, whereas none of the structures exhibited greater intensity levels for the 65-kDa band than the IC (Figure 4F). The superior colliculus and the cerebellum presented lower relative protein levels of the 104-kDa band than the IC (Figure 4F). Together, the western blotting results suggested different sets and levels of GluK1 proteins expressed among the structures involved in the seizure neuronal network of the GASH/Sal model, showing an unexpected lower molecular weight band in the inferior and superior colliculi.

### 3.4 Distribution of GluK1-immunoreactivity

**FIGURE 5:**
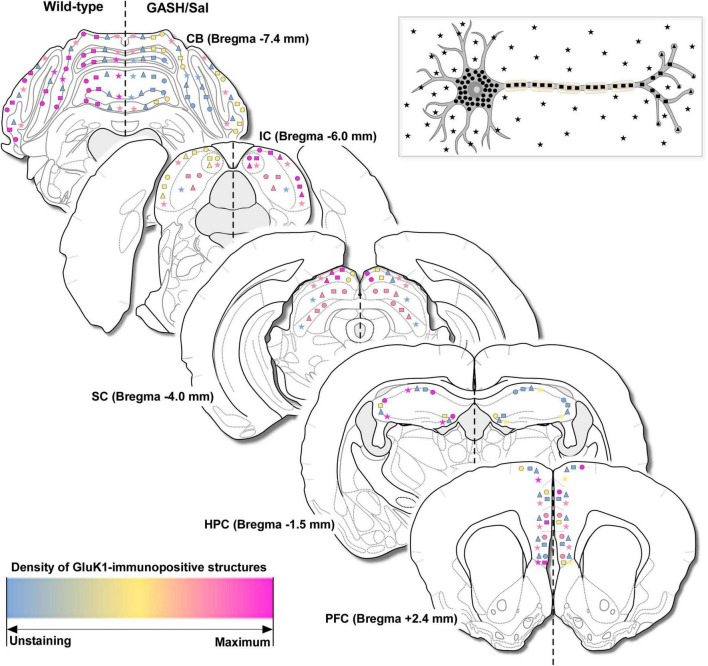
Overview of the immunohistochemical findings. Schematic representation of GluK1-immunopositive structures in the brain of the control and GASH/Sal hamsters corresponding to caudal-rostral levels (with respect to bregma) and based on visual qualitative observations. GluK1-immunoreactivity was categorized in cell bodies (circles), axonal fibers (rectangles), terminals (triangles) and punctate-like immunostaining nearby somata or in the neuropile (stars), according to the neuron diagram showed in the upper part of the figure. For comparative purposes, this classification was combined with a description of the density for GluK1-immunolabeling, ranging from unstained (in blue), to fewer (in yellow), similar (in pink), up to maximal immunostaining for numerous and dense immunoreactive structures (in magenta) as comparing one animal group to another. The information is provided in the left and right hemispheres for the wild-type and GASH/Sal hamsters, respectively. Bregma data from the hamster brain atlas of Morin and Wood (2001). CB, cerebellum; IC, inferior colliculus; SC, superior colliculus; HPC, hippocampus; PFC, prefrontal cortex.

**FIGURE 6:**
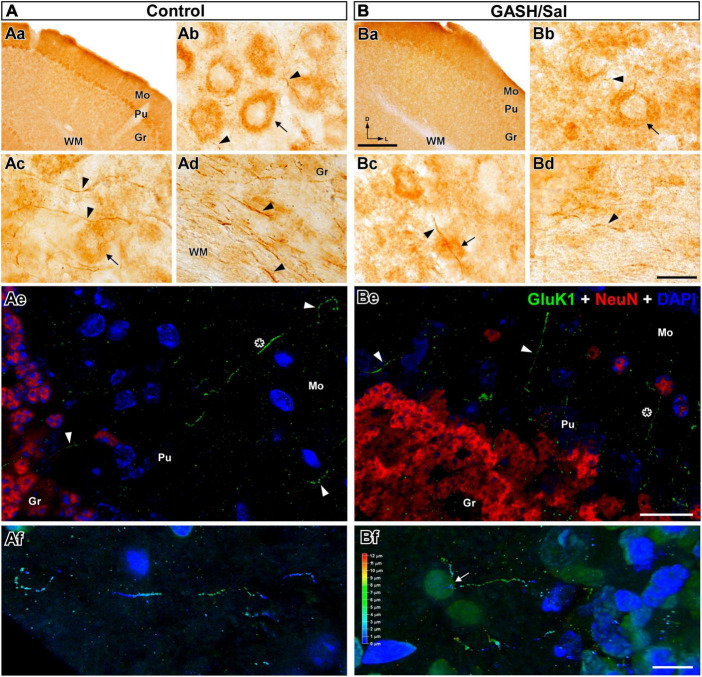
GluK1-immunolabeling in the cerebellum (CB) of the control **(A)** and GASH/Sal **(B)** hamsters. **(Aa,Ba)** Low magnification photomicrographs of representative coronal sections of the CB. Notice that Purkinje cells (Pu) were the cerebellar cell type most intensely immunolabeled for GluK1. GluK1-immunoreactivity was weaker in the GASH/Sal cerebellum as compared to controls. Photomicrographs showing details of GluK1-immunopositive cell bodies [arrow in **(Ab)** somatic size: 340.4 μm^2^ with major and minor axes of 24.1 and 17.9 μm; arrow in **(Bb)** somatic size: 456.5 μm^2^ with major and minor axes of 25.8 and 22.4 μm] and thin axonal fibers (arrowheads, diameter: ∼ 0.5 μm) in the CB. Notice large neuronal perikarya heavily immunolabeled for GluK1 in the Pu layer. No staining of neuronal nuclei and dendrites was observed with anti-GluK1. **(Ac,Bc)** GluK1-immunoreactive axonal fibers (arrowheads) passing in proximity and through Purkinje neuronal somata (arrow). **(Ad,Bd)** GluK1-immunoreactive axonal fibers (arrowheads: diameter: ∼ 0.7 μm) running into the white matter (WM) of the CB. Notice that fewer GluK1-immunopositive structures in the GASH/Sal cerebellum as compared to controls **(Ab–Bd)**. **(Ae,Be)** Confocal images show GluK1-immunopositive axonal fibers (in green, arrowheads) coursing into the three layers in the cerebellar cortex. DAPI (in blue) and the NeuN-immunolabeling (in red) were used to identify the cytoarchitecture of the brain nuclei. Purkinje cell layer was not labeled for NeuN. Notice the diffuse GluK1-immunolabeling in the cerebellar neuropile. **(Af,Bf)** Confocal micrographs of stereo projections corresponding to the area depicted with an asterisk in **(Ae,Be)** show details of GluK1-immunopositive fibers bifurcating in the cerebellar molecular layer (white arrow). Gr, cerebellar granular layer; Mo, cerebellar molecular layer; Pu, Purkinje cell layer. Scale bars = 200 μm in **(Aa,Ba)**; 20 μm in **(Ab–Be)**; 10 μm in **(Af,Bf)**.

**FIGURE 7:**
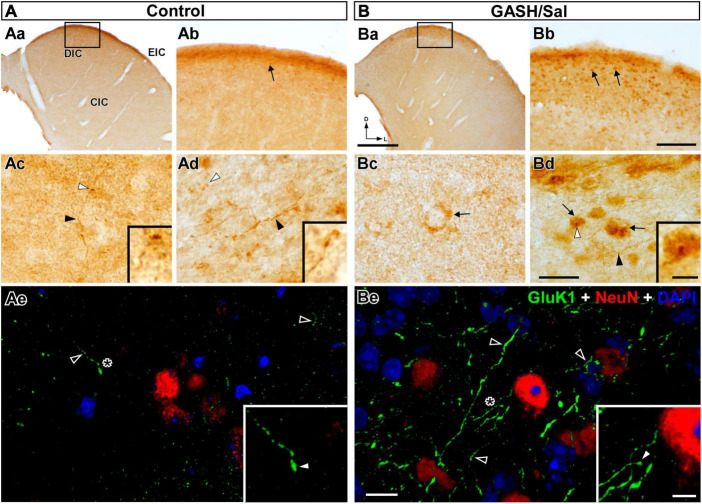
GluK1-immunolabeling in the inferior colliculus (IC) of the control **(A)** and GASH/Sal **(B)** hamsters. **(Aa,Ba)** Low magnification photomicrographs of representative coronal sections of the IC. Higher magnification of the dorsal cortex of the IC (DIC) corresponding to the frame in **(Aa,Ba)**. Notice greater number of GluK1-immunopositive neuronal somata (arrows; somatic size: ∼ 50 μm^2^ with major and minor axes of ∼ 9.5 and ∼ 6.8 μm) in the GASH/Sal as compared to controls. **(Ac,Bc)** Photomicrographs of the central nucleus of the IC (CIC) shows a GluK1-immunoreactive axonal fiber (black arrowhead) giving rise to an ending (white arrowhead) as well as diffuse GluK1-immunolabeling of neuronal somata (arrow). **(Ad,Bd)** High magnification photomicrographs of the external cortex of the IC (EIC) shows GluK1-immunopositive axonal fibers (black arrowheads) and endings (white arrowheads) in apposition to GluK1-immunopositive cell bodies (arrows). Insets show details of GluK1-immunopositive endings (corresponding to the white arrowheads in each panel). **(Ae,Be)** Confocal images of the DIC stained using anti-GluK1 (green), anti-NeuN (red), and DAPI (blue). Notice higher density of strong GluK1-immunopositive axonal fibers (black arrowheads) in the GASH/Sal as compared to controls. Insets show details of GluK1-immunopositive endings (white arrowheads) corresponding to the asterisk depicted in **(Ae,Be)**. The maximum projection of confocal images corresponding to the panels **(Ae,Be)** was displayed in the 3D video of Supplementary material 4. No staining of neuronal nuclei and dendrites was observed with anti-GluK1 in the IC. Scale bars = 500 μm in **(Aa,Ba)**; 100 μm in **(Ab,Bb)**; 20 μm in **(Ac,Bc)** and **(Ad,Bd)**; 10 μm in **(Ae,Be)**; and 5 μm for all insets.

**FIGURE 8:**
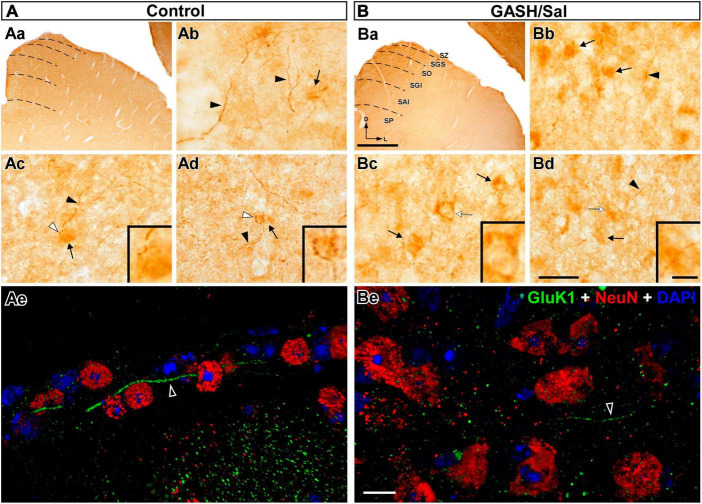
GluK1-immunolabeling in the superior colliculus (SC) of the control **(A)** and GASH/Sal **(B)** hamsters. **(Aa,Ba)** Representative photomicrographs of coronal sections of the SC show GluK1-immunolabeling in the superficial (SZ, SGS, and SO) and deep (SGI, SAI, and SP) subdivisions. Dashed lines indicate the collicular lamination. **(Ab,Bb)** Higher magnification of the external layers (SZ and SGS) show strong GluK1-immunoreactivity in axonal fibers (black arrowhead) of various thicknesses as well as diffuse immunoreactivity in the cell bodies (arrows). Notice fewer number of fibers and a greater number of GluK1-immunopositive cell bodies in the GASH/Sal as compared to controls. **(Ac,d,Bc,d)** Photomicrographs of the intermediate layers (SGI and SAI) show GluK1-immunopositive axonal fibers (black arrowheads) and endings (white arrowheads) in apposition to GluK1-immunopositive cell bodies (arrows). Insets show details of GluK1-immunopositive endings and somata (corresponding to the white arrowhead and arrow in each panel, respectively). Notice strong GluK1-immunopositive fibers give rise to axonal terminals, whereas in the GASH/Sal, there were no GluK1-immunopositive endings. Additionally, cell bodies in the GASH/Sal exhibited stronger GluK1-immunoreactivity. Punctate GluK1-immunolabeling was observed throughout the collicular neuropile, while no staining of neuronal nuclei and dendrites was detected with anti-GluK1. **(Ae,Be)** Confocal images of the superficial subdivision using anti-GluK1 (green), anti-NeuN (red), and DAPI (blue). Notice long axonal fibers with GluK1-immunoreactvity (black arrowheads) coursing throughout the collicular tissue and overcoming cell bodies. Notice weaker GluK1-immunolabeling in the axonal fiber of the GASH/Sal as compared to the wild-type animal. Punctate GluK1-immunolabeling was also observed in the neuropile. The maximum projection of confocal images corresponding to the panel **(Ae)** was displayed in the 3D video of Supplementary material 6. SAI, stratum album intermedium; SGI, stratum griseum intermediale; SGS, stratum griseum superficiale; SP, stratum profundum; SO, stratum opticum; SZ, stratum zonale. Scale bars = 500 μm in **(Aa,Ba)**; 20 μm in **(Ab–Bd)**; 10 μm in **(Ae,Be)**; and 5 μm for all insets.

**FIGURE 9:**
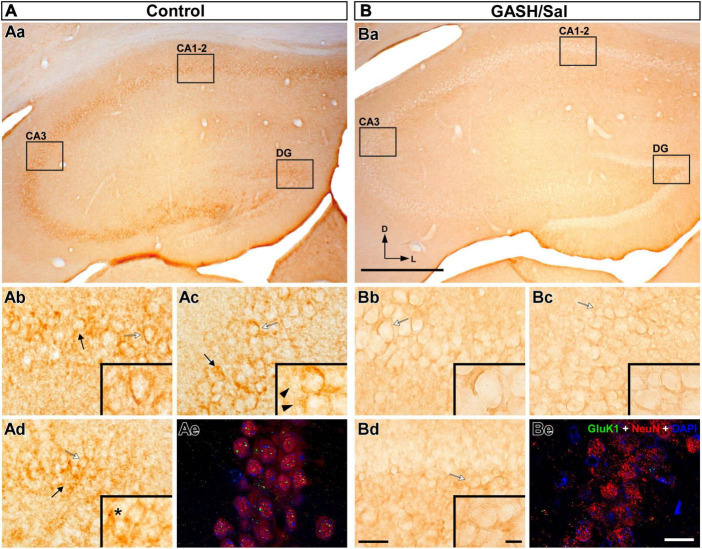
GluK1-immunolabeling in the hippocampus (HPC) of the control **(A)** and GASH/Sal **(B)** hamsters. **(Aa,Ba)** Representative low magnification images of the HPC, showing the three hippocampal regions. Notice the specific loss of GluK1-immunoreactivity in the hippocampal regions of the GASH/Sal. [High magnification photomicrographs of three hippocampal regions: CA1–2 **(Ab,Bb)**, CA3 **(Ac,Bc)**, and the dentate gyrus DG, **(Ad,Bd)**] corresponding to the black rectangles in **(Aa,Ba)**. Notice the strong GluK1-immunoreactivity in the cell bodies (black arrows) throughout the HPC of the control animal, whereas the GASH/Sal hippocampal regions show absence of GluK1-immunolabeling. The insets show details of GluK1-immunolabeling corresponding to the white arrow in each panel. Punctate GluK1-immunostaining (asterisk) and thin fibers (black arrowhead) immunolabeled for GluK1 were also observed in control animals. **(Ae,Be)** Confocal images of the CA3 region stained using anti-GluK1 (green), anti-NeuN (red), and DAPI (blue). Notice weaker punctate GluK1-immunolabeling in somata and neuropile of the GASH/Sal as compared to the wild-type animal. Scale bars = 500 μm in **(Aa,Ba)**; 40 μm in **(Ab,Bb)**, **(Ac–Bc,Ad,Bd)**; 20 μm in **(Ae,Be)**; and 10 μm for all insets.

**FIGURE 10:**
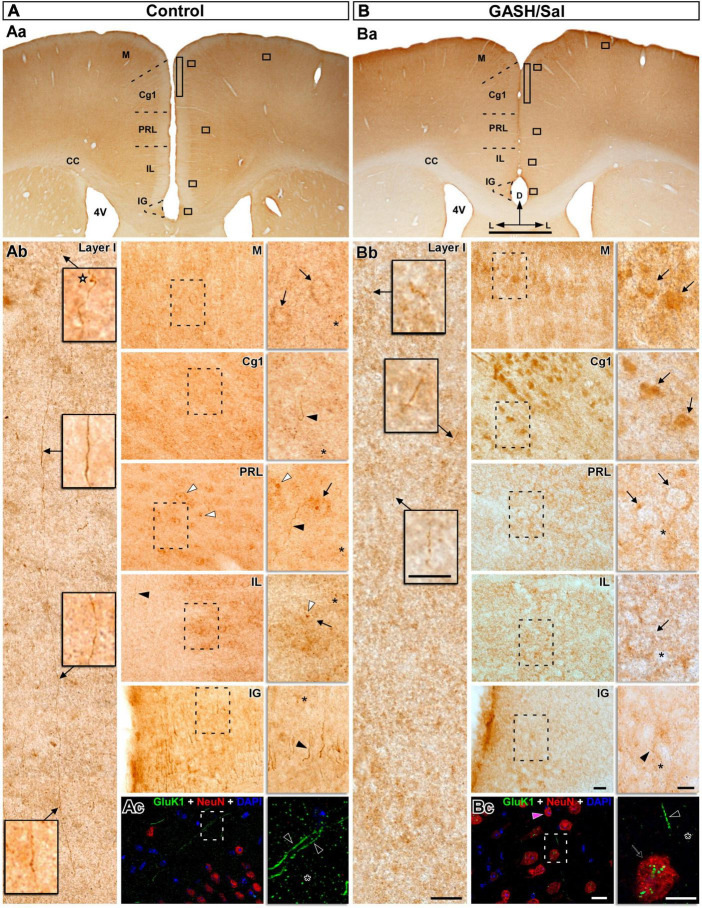
GluK1-immunolabeling in the prefrontal cortex (PFC) of the control **(A)** and GASH/Sal **(B)** hamsters. **(Aa,Ba)** Representative low-magnification images of coronal sections containing distinct cytoarchitectural regions of the medial PFC, including the cingulate cortex (Cg1), prelimbic cortex (PRL), and infralimbic cortex (IL), as well as adjacent areas such as the indusium griseum (IG) and the motor cortex (M). **(Ab,Bb)** High magnification photomicrographs corresponding to the squares in panels **(Aa,Ba)** show the GluK1-immunoreactivity pattern. Details of the selected brain areas corresponding to the dashed box are shown in zoomed-in images using 100x objective lens. Notice that strong GluK1-immunopositive fibers ran parallel to the long dorso-ventral axis through the layer I and give rise axonal terminals (star) in control animals, whereas the GluK1-immunopositive fibers were minimal in the GASH/Sal. Diffuse GluK1-immunopositive cell-like structures (arrows) were observed in M, PRL and IL areas as well as strong GluK1-immunopositive axonal fibers (black arrowheads) in the Cg1 and IG areas of control animals. In GASH/Sal animals, strong GluK1-immunoreactivity are found in the cell bodies (arrows) of the M and Cg1 areas, whereas GluK1-immunoreactive somata were low in PRL and IL areas. Low or absent immunoreactivity was detected in axonal fibers of the GASH/Sal (e.g., black arrowhead in the IG). Note the difference in the intensity of the punctate immunostaining (asterisk) between the control and GASH/Sal animals, as well as between the selected brain areas. GluK1-immunoreactivity concentrated nearby nucleus-like organelles (white arrowheads) in both animal groups (see Supplementary material 11 for additional examples). Confocal merged images showing localization of GluK1 (green), NeuN (red), and DAPI (blue) in the layer I-II **(Ac)** and layer V **(Bc)** of the Cg1 area. Numerous and long GluK1-immunopositive axonal fibers (black arrows) and punctate immunostained (asterisks) were observed in control animals, whereas fewer and lower GluK1-immunoreactivity was present in the GASH/Sal. Notice detail of somata-showing GluK1-immunopositive puncta (arrow) in the GASH/Sal. The maximum projection of confocal images corresponding to the panel 10Ac was displayed in the 3D video of Supplementary material 10. The orthogonal analysis of the neuron highlighted with a magenta arrowhead in the panel **(Bc)** is shown in the Supplementary material 12. Scale bars = 1 mm in **(Aa,Ba)**; 20 μm in **(Ab,Bb)**, and 10 μm for all insets.

We next investigated the bright-field and confocal microscopic immunohistochemical localization of the GluK1 receptor in the brain structures associated with the seizure neuronal network of the GASH/Sal as compared to wild-type animals. The overall immunostaining with antibodies to GluK1 was light to moderate in many structures throughout the brain of both animal groups, with a regional distribution in each brain area rather heterogeneous (Figure 5). In general, GluK1-immunoreactivity was diffusely distributed and often concentrated in neuronal perikarya, axonal fiber tracts, and terminals as well as a punctate-like immunostaining in the neuropile (Figure 5). At the resolution of optical microscopy, no clear immunostaining was observed in postsynaptic elements, including dendrites, spines, and dendritic shafts, which was an unexpected finding that will be further discussed below. Thus, morphological features that distinguished axonal fibers from dendrites such as the constant diameter for most of its length, the branching pattern and the area covered from the soma were taken into consideration in the histological analysis. In most cases, the diffuse immunoreaction product was not sufficient for identification of the GluK1-immunolabeled processes at low magnifications, just becoming clearly visible using high objective lenses. A qualitative evaluation of the immunohistochemical distribution of the GluK1 receptor in the five brain areas of study is summarized in Figure 5, categorizing the immunolabeling of somata and visible processes with a description of the density as comparing one animal group to another. Differences between the immunostaining result, the distribution and density of GluK1-immunolabeled structures were noted between the tissue sampled from the control and GASH/Sal animals. Representative bright-field and confocal images as well as the details on the GluK1-immunoreactivity in the brain areas of interest are depicted at low and higher magnifications of coronal sections in Figures 6–10. To provide a more comprehensive description of GluK1 receptor distribution, we have included additional representative examples and supporting information in Supplementary materials 4–12, including high-resolution confocal micrographs, orthogonal analysis, and 3D videos of maximum and stereo projections of confocal microscope image stacks.

#### 3.4.1 Cerebellum

The hamster cerebellum, like other rodent species, is a well-organized structure consisting of three distinct layers in its cortex: molecular, Purkinje cell, and granular layers, enabling the accurate coordination of movements and balance. In the cerebellum of control and GASH/Sal animals, conspicuous Gluk1-immunoreactivity was observed in neuronal perikarya, particularly in the Purkinje cell layer (Figures 6Aa, Ba). This immunoreactivity consisted of small and fine puncta, less than 1 μm in diameter, that cluster over the large cell bodies, presumably of Purkinje cells (Figures 6Ab, Bb). GluK1-immunolabeling was also evident within processes that were most likely to be axonal fibers following the criterion above mentioned. These fibers of varying calibers (0.2–0.5 μm in diameter) occurred in the vicinity of Purkinje neurons (Figures 6Ac, Bc). Those with such disposition and very thin diameter claims be identifiable as cerebellar parallel fibers. Axonal processes or fibers bundles were also intensely immunolabeled for GluK1 coursing throughout the white matter of the cerebellum (Figures 6Ad, Bd). At the confocal microscopy, GluK1-immunopositive axonal fibers were also detected through the layers of the cerebellar cortex, which were distinguishable through NeuN-immunostained and DAPI labeling (Figures 6Ae, Be). These GluK1-immunolabeled axons appeared coursing from the granular cell layer, ascending through the Purkinje cell layer to the lower molecular layer, in which the dendritic trees of the Purkinje neuron presumably distributed (Figures 6Ae, Be). The stereo view of confocal image sections showed that these GluK1-fibers bifurcated in the molecular layer as typically occurred in parallel fibers (Figures 6Af, Bf). Together, these results suggest that GluK1-immunoreactivity was intense in the cerebellar regions containing parallel fibers-Purkinje cells synapses. In addition, GluK1-immunoreactivity was observed as diffuse immunostaining of neuropile in the cerebellar cortex (Figures 6Ae, Be). As compared to controls, the cerebellum of the GASH/Sal exhibited less intensity of GluK1-immunoreactivity in the neuronal profiles (Figures 6A, B). The GluK1-immunopositive structures were less homogeneously distributed in the GASH/Sal, showing cerebellar lobules with absent or weak immunoreactivity (Figure 5). The number of cell bodies and axonal fibers immunolabeled for GluK1 were reduced in the GASH/Sal cerebellum, while the labeling of neuropile was seen similarly in diverse cerebellar regions (Figures 5, 6).

#### 3.4.2 Inferior colliculus

Located in the midbrain, the IC is a vital paired structure serving as a relay center for auditory information and other non-auditory sources, with its main regions being the central nucleus, dorsal cortex, and external cortex. The distribution of GluK1-immunoreactivity in the IC was found to be heterogeneous in both animal groups, exhibiting variations across the three subdivisions. The observed density and intensity of the immunoreactive product was higher in the dorsal and external cortex compared to the central nucleus of the IC (Figures 5, 7Aa, Ba). In the dorsal and external cortex, the GluK1 distributed within small neuronal perikarya (Figures 7Ab, Bb) as well as axonal fibers that generate boutons near cell bodies (Figures 7Ad, Bd). While similar immunolabeled structures were observed in the central nucleus, the immunostaining was diffuse and weakly distributed, particularly in scarce cell bodies (Figures 7Ac, Bc). Using confocal microscopy, we observed specific GluK1-immunostaining in axonal fibers as well as in the neuropile in the external regions of the IC. These long fibers gave rise to endings on cell bodies, which we visualized with NeuN-immunostaining (Figures 7Ae, Be). Supplementary material 4 includes a 3D video with split-channel visualization of maximum projection of confocal images, providing a close-up view of the GluK1-immunopositive axonal fibers and endings. Upon comparison of GASH/Sal and controls, clear and significant differences were observed (Figure 5). In the external cortex, bright-field and confocal microscopic examination revealed a higher density of GluK1-immunopositive cell bodies and axonal fibers, with much more numerous axonal branches and endings in the GASH/Sal group compared to controls (Figures 5, 7Ae, Be and Supplementary material 4). Although GluK1-immunolabeled structures were sparse in the central nucleus in both groups, there was a slightly higher density of GluK1-immunolabeled cell bodies in the GASH/Sal group compared to controls, while GluK1-immunolabeled axonal fibers and terminals were slightly more prominent in the control group (Figures 5, 7). Interestingly, GluK1-immunoreactivity was notably concentrated around the DAPI-stained nucleus with a higher frequency observed in the GASH/Sal group, especially within the external cortex, as vividly depicted in orthogonal confocal views (Supplementary material 5). In summary, the observed marked differences in GluK1-immunoreactivity between the regions of the IC in GASH/Sal animals suggest potential underlying glutamate synaptic transmission alterations that may be related to the role of the IC in seizure generation and propagation.

#### 3.4.3 Superior colliculus

Neuroanatomically, the superior colliculus is comprised of a highly organized, six-layer structure. The three superficial layers are primarily responsible for receiving visual information, whereas the three deeper layers are interconnected with cortical and subcortical areas involved in auditory, somatosensory and motor function. Thus, GluK1-immunolabeling was compared between control and GASH/Sal hamsters, considering these two functionally distinct units, a superficial subdivision comprising the stratum zonale, stratum griseum superficiale, and stratum opticum, and a deep subdivision comprising stratum griseum intermediale, stratum album intermedium, and stratum profundum (Figure 8). The superficial subdivision exhibited robust GluK1-immunoreactivity in axonal fibers of varying thicknesses, as well as diffuse immunoreactivity in cell bodies in both animal groups (Figures 8Ab, Bb). Additionally, the GASH/Sal group displays a lower number of fibers and a higher number of GluK1-immunopositive cell bodies when compared to the control group (Figures 5, 8Ab, Bb). The deep subdivision, particularly the intermediate layers, exhibited GluK1-immunopositive axonal fibers that terminate in close proximity to GluK1-immunopositive cell bodies. In the wild-type animal, these fibers give rise to axonal terminals that are strongly GluK1-immunopositive (Figures 8Ac, Ad). However, a remarkable finding in the GASH/Sal animal was the absence of GluK1-immunopositive axonal terminals, despite exhibiting stronger GluK1-immunoreactivity in cell bodies (Figures 5, 8Bc, Bd). Although the deepest layers (striatum profundum) exhibited very weak immunostaining, punctate GluK1-immunolabeling was observed throughout the collicular neuropile in both animal groups (e.g., Figure 8Ad). All these findings agreed with those observed at confocal microscopy, which consistently revealed the presence of long axonal fibers exhibiting GluK1-immunoreactivity coursing throughout the external layers, passing over neuronal perikarya stained with anti-NeuN (Figure 8Ae and Supplementary material 6). Notably, the axonal fibers, including those observed through the dorso-ventral axis of the collicular lamination, exhibited weaker GluK1-immunolabeling in the GASH/Sal animal when compared to those of the control animal (Figures 8Ae, Be). Due to the critical role played by the superior colliculus in integrating diverse sensory modalities to generate appropriate behavioral responses and transforming this sensory information into motor commands, the observed differences in Gluk1-immunostaining between the two animal groups underscore its significance for seizure propagation in the GASH/Sal model.

#### 3.4.4 Hippocampus

The hippocampus, a major limbic region in the brain, is composed of several subregions, including the dentate gyrus, CA1, CA2, and CA3 regions, which are interconnected via unidirectional pathways. As expected, strong Gluk1-immunoreactivity decorated the numerous cell bodies in the three hippocampal regions of the wild-type animals (Figure 9Aa). This immunostaining was found homogeneously distributed between the hippocampal regions as well as the rostro-caudal axis of the hippocampus. At higher magnification, the GluK1-immunostaining shows a characteristic subcellular distribution, with a prominent accumulation near the cell membranes of the somata, forming small and distinct punctate structures, as illustrated in Figures 9Ab–d as well as Supplementary materials 7, 8. Notably, the thin axons (with an approximate diameter of 1.5 μm) were found to be sparse in the hippocampal regions and were rarely detected in CA3 and dentate gyrus (Figure 9Ac and Supplementary materials 7, 8). In line with our findings in other brain regions, no dendrites were found to exhibit immunoreactivity with the anti-GluK1 antibody (Figures 9Ab–d). A comparison with control animals revealed a striking contrast in the hippocampus of the GASH/Sal animals, where a marked reduction in GluK1-immunostaining was observed (Figures 5, 9Ba). All hippocampal regions of the GASH/Sal animals displayed a remarkably weak, almost negligible level of GluK1-immunoreactivity (Figures 5, 9Bb–d), indicating a potential disruption in the expression or localization of this protein in the GASH/Sal model. In particular, the dentate gyrus, a relevant hippocampal area during seizures, exhibited a notable absence of strong GluK1 punctate-immunostaining in the neuronal perikarya of GASH/Sal animals, which contrasted with the observation in the wild-type animals (Supplementary material 7). Consistent results were obtained from both bright-field and confocal microscopy techniques, as a decrease in GluK1 punctate-immunolabeling was observed in the somata and neuropile of GASH/Sal animals. Notably, the confocal microscopy analysis showed that GASH/Sal animals exhibited sparse immunofluorescence for the GluK1 receptor in contrast to the control animals, which showed robust punctate GluK1-immunolabeling in the same regions (Figures 9Ae, Be and Supplementary material 7). Despite the weak immunolabeling of GASH/Sal hippocampus, the confocal microscopy analysis revealed that GluK1-immunostaining was gathered around the DAPI-stained nucleus, as observed in close-up orthogonal views in the dentate gyrus (Supplementary material 9). These findings suggest an alteration in the localization of GluK1 protein in the hippocampus, a limbic structure that indirectly receives auditory signals from the brainstem, which may be relevant in understanding the limbic recruitment during repeated audiogenic seizures in the GASH/Sal model.

#### 3.4.5 Prefrontal cortex

The distribution of GluK1 was examined in sections taken from the middle portions of the prefrontal cortex, which encompass several discernible cytoarchitectural regions, including the cingulate cortex, prelimbic cortex, and infralimbic cortex, as well as neighboring areas such as the indusium griseum and the motor cortex. In control animals, the labeling for GluK1 was relatively heterogenous across the cortical laminae and made up of small puncta in the neuropile, axonal fibers, terminals, and perikarya (Figure 10A). At higher magnification, GluK1-immunoreactivity was seen in several compartments across the different regions of the prefrontal cortex (Figure 10Ab). In the cingulate cortex of control animals, we observed robust GluK1-immunopositive fibers that give off terminals, as well as abundant small puncta in the neuropile. Long axonal fibers, which were notably marked for GluK1, were observed coursing through the dorsoventral axis of layer I and giving off terminals (e.g., in Figure 10Ab). Diffuse punctate granular pattern of neuropile staining with labeling of neuronal cell bodies was observed in the prelimbic and infralimbic areas as well as strong GluK1-immunopositive axonal fibers in the cingulate cortex areas of control animals (Figure 10Ab). Similarly, strong axonal fibers were found in the indusium griseum, and diffuse labeling was detected in the cell bodies of the motor cortex (Figure 10Ab). As compared to controls, GASH/Sal animals exhibited strong GluK1-immunoreactivity in the cell bodies distributed in the motor and cingulate cortex (Figure 10Ba), while the prelimbic and infralimbic regions showed similar immunoreactivity in neuronal perikarya (Figures 5, 10Bb). Notably, axonal fibers of the GASH/Sal animals showed little to no immunoreactivity in the isidium griseum (Figure 10Bb). Furthermore, there was a marked difference in the intensity of the punctate immunostaining observed between the control and GASH/Sal animals, as well as between the selected brain regions. As in other examined brain areas, it is worth noting that the GluK1-immunoreactivity was found to be concentrated in the vicinity of nucleus-like organelles in both animal groups, but more frequently observed in the GASH/Sal (Figures 10A, B and Supplementary materials 11, 12). Consistent with the bright-field microscopy observations, confocal image analysis revealed that the same pattern of immunolabeling was observed in control animals, with numerous and lengthy GluK1-immunopositive axonal fibers as well as neuropile immunolabeling in the form of a fine dusting in the medial prefrontal cortex regions (Supplementary material 10). In the GASH/Sal animals, both microscopy techniques showed fewer and less intense GluK1-immunoreactivity in the same regions, and a closer examination revealed the presence of GluK1-immunopositive puncta weaky labeled in the somata and neuropile (see for comparison Figures 10Ac, Bc and Supplementary materials 11, 12). In sum, these results pointed out an altered distribution in GluK1 receptors that could lead to an increase in neuronal excitability and potentially alter synaptic plasticity in the medial prefrontal cortex of the GASH/Sal model.

## 4 Discussion

The GASH/Sal strain was previously analyzed using whole exome sequencing to identify and characterize its mutational landscape (Díaz-Casado et al., 2020). Moderate- and high-impact variants were validated using Sanger sequencing, including a single amino acid substitution from histidine to tyrosine in the GluK1 protein (Díaz-Casado et al., 2020). The impact of the p.H289Y polymorphism on the lifespan of the GluK1 protein and its potential effects on the function of the glutamatergic system in the GASH/Sal brain, if any, pose highly intricate and multifaceted inquiries. The present study explores the effects of this single-nucleotide polymorphism on brain structures involved in the neuronal network associated with audiogenic seizures in the GASH/Sal model. Through a predicted 3D model, we found that this missense mutation affects protein stabilization by increasing intermolecular interactions. Gene expression analysis revealed disturbances in the *Grik1* gene’s transcriptional profile within the neuronal network associated to audiogenic seizures. Furthermore, variations in GluK1 protein patterns and levels were observed heterogeneously among the brain structures, including an unexpected lower molecular weight band in the inferior and superior colliculi. Immunohistochemical examination of GluK1 receptor distribution across the audiogenic seizure neuronal network revealed significant differences in GluK1-immunoreactivity, suggesting potential alterations in glutamate synaptic transmission in the GASH/Sal model. Overall, this study deepens our understanding of genetic alterations in seizure neural networks, particularly regarding the role of kainate receptors.

### 4.1 Potential protein stabilization caused by the p.H289Y polymorphism in the *Grik1* gene

To understand the functional and disease implications of genetic variations, it is crucial to have knowledge of the 3D structure of the gene product. In the context of utilizing predicted structures, such as in the present study, the question arises as to whether the predictions derived from a 3D model align with those obtained from an experimental structure and how the accuracy of the models impacts these findings. Nonetheless, evidence exists to support the notion that predicted protein 3D models are just as effective as experimental structures in determining the pathogenic impact of a variant (Ittisoponpisan et al., 2019). Changes at the amino acid level can affect the protein’s shape, folding, function, stability, ligand binding properties, catalysis, regulation, and post-translational modification, ultimately impacting the phenotype. While the structural context of each missense mutation can predict likely changes in molecular function, it is important to consider potential caveats, such as mutations that may be neutral in terms of phenotype rather than molecular function and effects that cannot be easily identified from structure alone (Wang and Moult, 2001). Therefore, each mutation can affect the various roles of a residue through modification of different types of interactions, including hydrophobic, hydrogen bond, van der Waals, electrostatic interactions, and disulfide bonds (Wang and Moult, 2001). Taking this into consideration, our protein structure analysis revealed the emergence of new hydrophobic-proximal interactions resulting from the p.H289Y genetic variant, which differs from the wild-type structure, implying the likelihood of alterations in the intermolecular interactions within the GluK1 subunit. Histidine and tyrosine are distinct amino acids that differ significantly in their chemical structures and properties. Tyrosine, which possesses a large amphipathic side chain that can participate in non-polar, hydrogen-bonding, and cation-π interactions, is highly effective in mediating recognition in protein-protein interfaces in contrast to histidine, which lacks these features (Koide and Sidhu, 2009). However, considering its location in the ATD rather than the binding domain, the p.H289Y genetic variant is unlikely to directly compromise protein binding with glutamate. Consequently, it is anticipated to exert only a minimal or negligible effect on this activity. This leads us to argue on the potential implications of the substitution of histidine-289 to tyrosine in the ATD, specifically in the regulatory region R2, of the GluK1 receptor. One feasible possibility is that this point mutation may affect its assembly into tetramers from the pool of the five kainate receptor subunit types. Supporting this, research has shown that high-affinity interactions in the ATD are necessary for the biosynthesis of functional heteromeric kainate receptors (Ayalon and Stern-Bach, 2001; Kumar et al., 2011), and that the GluK1 subunit’s ATD can form heteromeric receptors with all possible stoichiometries in an expression-dependent manner (Selvakumar et al., 2021). Although the inflexibility of the lower lobe (R2) interaction within the ATD is thought to be the cause of the lack of allosteric regulation in kainate receptors (Furukawa, 2012; Gangwar et al., 2023), the possibility that the p.H289Y polymorphism may impact this aspect cannot be dismissed, as the ATD contains extensive interactions with the ligand binding domains that allow for complex allosteric regulation, as observed in other ionotropic glutamate receptor subunits (Krieger et al., 2015). Interestingly, Matsuda et al. (2016) showed that complement proteins specifically bound the ATD of kainate receptors subunits, serving as master regulators of postsynaptic kainate receptor complexes. Although the role of the complement system in the GASH/Sal model remains unexplored, the dysregulation of complement proteins contributing to disease progression during synaptic pruning in development and disease (Stephan et al., 2012), underscores the importance of further exploring this potential influence. In line with this notion, the complement system has been implicated in the pathogenesis of epilepsy and may provide opportunities for developing better therapeutics and prognostic markers (Kopczynska et al., 2018). Duan et al. (2018) uncovered a trafficking mechanism for kainate receptors, suggesting that the cleaved signal peptide acts as a ligand for GluK1, binding with the ATD to repress receptor trafficking. Thus, a single point mutation in this domain could disrupt this crucial molecular function, leading to an alteration in the forward trafficking ability of the GluK1 receptor. Finally, our study employs several stability predictors to assess folding or protein interaction energy changes upon this mutation. The collective and convergence findings from all stability predictors utilized in our analysis consistently indicate that the p.H289Y polymorphism presumably leads to significant protein stabilization. Furthermore, the observed increase in total interactions within the protein carrying the p.H289Y genetic variant, compared to the wild-type structure, aligns with the predicted over-stabilization of the mutated GluK1 protein. Given the substantial amino acid sequence similarity of GluK1 protein among various species, as noted in Supplementary material 1D, it is plausible to extrapolate that the anticipated effect of this specific point mutation could be analogous. Computational stability predictors, such as those employed in our study, have become a widely adopted approach to assess the impact of missense mutations by evaluating changes in protein folding and interaction energy (Gerasimavicius et al., 2020). Although stability predictors were not specifically designed to identify pathogenic variants, their outputs are frequently included as supporting evidence for pathogenicity in publications reporting novel variants (Gerasimavicius et al., 2020). It is worth noting that although the previous emphasis was primarily on destabilizing mutations, it is now recognized that certain pathogenic missense mutations can stabilize protein structure. For instance, the histidine-to-glutamine substitution in the flexible loop of the chloride intracellular channel 2 (CLIC2) protein, which is associated with calcium ion signaling and epilepsy, is predicted to enhance protein stability (Takano et al., 2012). This prediction is supported by ΔΔG calculations indicating reduced flexibility and movement in the ATD as well as decreased membrane integration of the protein (Takano et al., 2012). In line with this rigidification argument, the heightened stability of the GluK1 protein’s ATD could potentially result in adverse effects. Although the specific implications of increased stability depend on the protein’s overall structure, function, and the context in which it operates, excessive stabilization of a protein might also prolong its half-life, elevate cellular concentration, and render it more resistant to denaturation or degradation. In the case of the GluK1 receptor, the over-stabilization of its ATD can affect folding, conformational dynamics, flexibility, and accessibility. This, in turn, may disrupt the intricate combinations of heteromeric kainate subunits and consequently impact the precise modulation of kainate receptor function within the GASH/Sal brain. Thus, the broader consequences of increased stability must not be disregarded, as they are closely intertwined with the protein’s overall structure, function, and operational context.

### 4.2 Dysregulated *Grik1* gene expression within the GASH/Sal seizure network

The next step in exploring the potential consequences of a stability-altering point mutation was to examine the expression pattern of the *Grik1* gene in brain structures associated with the seizure neuronal network in the GASH/Sal model. Previous reports from our research group have identified alterations in the gene expression of the GASH/Sal under free-seizures condition and after audiogenic seizures (López-López et al., 2017; Prieto-Martín et al., 2017; Damasceno et al., 2020; Díaz-Casado et al., 2020; Díaz-Rodríguez et al., 2020; Sánchez-Benito et al., 2020; Fuerte-Hortigón et al., 2021). Examining baseline alterations in gene expression provides insights into inherent changes contributing to the epileptic phenotype of the GASH/Sal, enabling the identification of genes involved in seizure predisposition and the overall pathophysiology. Moreover, analyzing gene expression after seizures reveals immediate molecular changes associated with seizure activity, like the seizure propagation pathways. The current study was conducted in GASH/Sal animals under free-seizures condition, showing significant differences in the mRNA expression levels of the *Grik1* gene between the GASH/Sal model and controls. Specifically, the cerebellum as well as the inferior and superior colliculi showed significantly higher expression of *Grik1* in the GASH/Sal, while the hippocampus exhibited downregulation. The consequences of dysregulation of the *Grik1* gene in these brain structures can vary depending on the context and the specific functions of that structure. Sánchez-Benito et al. (2020) investigated naïve GASH/Sal animals and discovered notably alterations in cochlear mechanotransduction, as evidenced by abnormal expression patterns in essential cochlear genes, including the vesicular glutamate transporters 1 and 2. Furthermore, they reported disrupted gene expression of these vesicular glutamate transporters in the cochlear nucleus. This observation hints at a disturbance in the normal glutamatergic transmission along the primary acoustic pathway, carrying significant implications for the potential propagation of abnormal transmission to the GASH/Sal inferior colliculus (Sánchez-Benito et al., 2020). As in other rodent models of epilepsy, seizure susceptibility corrupts acoustic integration in the IC, a critical structure involved in the initiation propagation of audiogenic seizures (Ribak and Morin, 1995; Pinto et al., 2019). Hence, our findings indicating the overexpression of *Grik1* in the IC suggest a potential contribution to heightened excitatory signaling and the potential disruption of neuronal activity balance, even in the absence of seizures. Furthermore, our results revealed that the *Grik1* gene expression was higher in the cerebellum and superior colliculus compared to the epileptogenic focus of the GASH/Sal model. This observation is noteworthy considering the role of the IC in transmitting epileptogenic events primarily through the deep layers of the superior colliculus to the motor nuclei of the brainstem (Faingold and Casebeer, 1999; Fuentes-Santamaría et al., 2007). Consequently, our findings have implications for sensory-motor integration pathways, potentially involving the cerebellum (McCandless and Schwartzenburg, 1982; Streng and Krook-Magnuson, 2021). Glutamate signaling, known for its significant dysregulation in epilepsy, was prominently characterized by layer-wise transcriptional alterations in multiple glutamate receptor genes (Pfisterer et al., 2020). Notably, newly identified genes including those encoding kainate receptor subunits and several AMPA auxiliary proteins, like the shisa9/cysteine-knot AMPAR modulating protein of 44 kDa (SHISA9/CKAMP44), were found dysregulated in epilepsy (Pfisterer et al., 2020). Consistent with these findings, the recent report by García-Peral et al. (2023) identified an altered expression of the SHISA9 protein within the IC of the GASH/Sal model. SHISA9, which is vital for proper brain development, function, and hearing, plays a crucial role in regulating glutamate receptor activity and synaptic transmission strength, potentially participating in tinnitus perception (Bhatt et al., 2022). Tinnitus and hyperacusis has been associated with hyperexcitability and increased spontaneous activity in auditory regions, including the cochlear nucleus (Auerbach et al., 2014) and the IC (Bauer et al., 2008). Consequently, the GASH/Sal model exhibits the morphological and molecular alterations that correlated with this hearing impairments as well as an inherited susceptibility to audiogenic seizure (Sánchez-Benito et al., 2017, 2020). Our results further suggest that the altered gene expression of *Grik1* may be a key factor contributing to the molecular correlates observed in the GASH/Sal model that led to elevated hyperactivity, enhanced synchrony, and disrupted neurotransmitter balance within seizure neuronal network. Indeed, the expression of immediate-early genes, which is part of the general neuronal response to natural stimuli, has been linked to the instability of homeostatic plasticity in tinnitus within the IC (Hu et al., 2014). This finding is consistent with our previous reports, demonstrating the overexpression of immediate-early genes in the IC of two genetically rodent models of audiogenic seizures (López-López et al., 2017; Damasceno et al., 2020; Díaz-Rodríguez et al., 2020). Certainly, the dysregulation of gene expression that influences the imbalance of neurotransmitters in the epileptogenic focus and the associated seizure neuronal network in the GASH/Sal is far more intricate. For instance, the cannabinoid system has been identified as playing a role in the regulation of auditory stimuli in the IC (Valdés-Baizabal et al., 2017). Additionally, glutamate can control inhibitory synaptic transmission through the simultaneous activation of presynaptic GluK1-containing kainate receptors and cannabinoid type 1 receptors (Kano et al., 2009; Lourenço et al., 2010). Therefore, it is plausible that any gene expression disruptions in those receptors could affect neuronal excitability. In a recent study, Fuerte-Hortigón et al. (2021) reported lower gene expression of cannabinoid type 1 receptors in the GASH/Sal inferior colliculus, as well as higher expression in the GASH/Sal hippocampus compared to wild-type counterparts. These findings, combined with our own observations of lower gene expression of *Grik1* in the GASH/Sal inferior colliculus and hippocampus, support the loss of this intrinsic synaptic modulation, contributing to the molecular basis of audiogenic seizure susceptibility. Notwithstanding, further investigations are necessary to understand the exact mechanisms by which dysregulation of kainate receptors and secondary neuromodulatory agents like the endocannabinoid system may alter neurotransmitter release in the GASH/Sal inferior colliculus and associated brain regions.

### 4.3 Alterations in Gluk1 protein levels within the GASH/Sal seizure neuronal network

Gene expression alterations do not always correlate directly with changes in protein levels due to the complex interplay of post-transcriptional, translational and protein degradation mechanisms (Vogel and Marcotte, 2012). Therefore, our study took a dual approach, examining both mRNA and protein levels, to thoroughly explore the implications of the *Grik1* polymorphism within the seizure neuronal network of the GASH/Sal model. Western blot analysis proved invaluable in exploring the molecular disparities between genetic rodent models of epilepsy and their wild-type counterparts, particularly for investigating altered protein abundance in specific brain regions (Bosque et al., 2021). Using the immunoblotting technique, we observed significant changes in GluK1 protein levels within the seizure associated structures of the GASH/Sal model. This finding prompts us to consider the interaction between GluK1 receptors and K^+^/Cl^–^ cotransporters, in order to shed light on the potential impact of GluK1-protein level disruption. It is well-established that subunits of kainate-type glutamate receptors, including GluK1 and GluK2, play a crucial role in the oligomerization and surface expression of K^+^/Cl^–^ cotransporters (Mahadevan et al., 2014). Furthermore, recent studies on the GASH/Sal strain have reported reduced levels of cation-chloride cotransporters NKCC1 and KCC2 in epileptic brain regions, both at rest and following repeated sound-induced seizures (Prieto-Martín et al., 2017; Bonet-Fernández et al., 2023). These authors suggested impaired functionality of the GABAergic system in the GASH/Sal model, as neuron-specific K^+^/Cl^–^ cotransporters NKCC1 and KCC2 are pivotal for maintaining low intracellular Cl^–^ levels, which are essential for facilitating rapid inhibitory synaptic transmission in the normal central nervous system (Prieto-Martín et al., 2017; Bonet-Fernández et al., 2023). Considering these facts, our observation of altered GluK1-protein abundance may indeed influence K^+^/Cl^–^ cotransporters, potentially disrupting the delicate balance between inhibition and excitation, thus contributing to the heightened susceptibility of the GASH/Sal model to audiogenic seizures. An intriguing finding in our study was the presence of two distinct bands in the inferior and superior colliculi of the GASH/Sal model: the expected band size of ∼104 kDa and an additional band at ∼65 kDa. A well-established phenomenon is that alternative splicing in transcripts significantly enhances proteome diversity by enabling a single gene to produce multiple distinct isoforms. In this context, if the splicing events result in different protein isoforms that retain the epitope recognized by the antibody, then Western blotting can indeed show multiple bands corresponding to these isoforms. Our multiple sequence alignment confirmed that the GluK1-antibody we used in our study effectively recognized a highly conserved antigenic region across all isoforms in hamsters, and hence, the antibody can bind to each isoform, leading to distinct bands on the blot. Notably, our results in the GASH/Sal contrasts sharply with those obtained in wild-type animals, where the 65-kDa band consistently remained absent across all brain structures. This stark difference suggests that the presence of the 65-kDa band in the GASH/Sal collicular structures may likely be attributed to a splicing variant unique to the GASH/Sal strain. Nevertheless, verifying this hypothesis will require empirical investigation, as other factors, such as post-translational protein modifications and the regulation of protein degradation, can also contribute to the presence of multiple bands in Western blots. Regarding kainate receptors, alternative splicing significantly enhances the functional spectrum of GluK1 receptors as these splice variants differ in their tissue-specific expression, membrane delivery processes, and protein interactions (Jaskolski et al., 2004). Therefore, our observations gain significance considering recent studies suggesting that splicing events affecting the ATD of GluK1 receptors have the potential to impact receptor assembly, stability, and modulation by interacting with the corresponding auxiliary proteins neuropilin and tolloid-like (Neto) (Dhingra et al., 2023).

### 4.4 Abnormal distribution of Gluk1 receptors within the GASH/Sal seizure neuronal network

In line with the established workflow for comprehensively studying protein biology in genetic rodent models of epilepsy (Bosque et al., 2021), our study extended its scope by conducting an immunohistochemical analysis of the GluK1 protein in the hamster brain. Thus, we provided crucial spatial and anatomical information about GluK1 distribution within the GASH/Sal seizure neuronal network. Interpreting the GluK1-immunolabeling pattern in hamster brain tissue posed challenges, primarily because these immunolabeled structures only became discernible at high magnifications. The scattered and faint GluK1-immunolabeling could, in part, be attributed to the section thickness (40 μm), potentially affecting staining penetration and the sensitivity for identifying individual processes. Thus, to ensure accurate interpretation, our study utilized a combined analysis, using both bright-field and confocal microscopes. The confocal fluorescence microscopy enabled us to capture thin sections of the sample and acquire a series of images at multiple discrete focus levels, providing a 3D view of the samples that was subsequently presented in videos as in previous studies (Gómez-Nieto et al., 2008b). We observed diffuse GluK1-immunoreactivity spread throughout the hamster brain, with concentrations in subcellular compartments such as neuronal perikarya, axonal fiber tracts, and terminals. Surprisingly, we were unable to identify clearly postsynaptic elements, including dendrites, spines, and dendritic shafts, that were expected to contain GluK1 receptors (Vesikansa et al., 2012). This does not conclusively exclude the possibility of GluK1 receptors being present in postsynaptic elements within the hamster brain. Some of the diffuse punctate immunostaining in the neuropile may indeed indicate immunostaining of both postsynaptic elements and axons, a phenomenon that can be associated with the sectioning process during brain tissue preparation. Achieving a clearer view of these postsynaptic structures may require the use of higher-resolution imaging techniques like transmission electron microscopy that can confirm their identity and offer valuable insights into the distribution of GluK1 receptors in these subcellular regions. An additional constraint in our study arises from the presence of GluK1 receptors in glial cells, including astrocytes, as reported by Eyigor et al. (2005) and Vargas et al. (2013). We did not employ a specific marker for glial cells, which leaves open the possibility that some of the staining observed in the neuropile may have also encompassed glial cells. Indeed, as demonstrated by Vargas et al. (2013) in an immunohistochemistry study, reactive hippocampal astrocytes were shown to express GluK1 subunits in a chemically induced temporal lobe epilepsy model. In this model, astrocytes initiated the expression of kainate receptors in response to seizure activity (Vargas et al., 2013). Thus, it may be worthwhile to investigate a similar phenomenon in GASH/Sal animals following repeated seizure stimulations or under kindling conditions.

The pattern of GluK1-immunoreactivity observed in the wild-type hamster brain in our study closely resembles that reported in brain areas of other mammalian species, with certain disparities also noted. As in our results, GluK1-immunolabeling decorating neuronal perikarya has been reported in the arcuate nucleus, hypothalamus, and dorsal cochlear nucleus of the rat (Diano et al., 1998; Eyigor et al., 2005; reviewed in Wu and Tang, 2023) as well as in the neocortex and hippocampus of dogs and monkeys (Good et al., 1993; Huntley et al., 1993; Hof et al., 1996). Furthermore, a notable finding in hamsters was the robust GluK1-immunolabeling present in presynaptic terminals, including axonal fibers and terminals. This observation stands in contrast to previous studies on the immunocytochemical localization of GluK1 receptors, where antibodies recognizing all low-affinity kainate receptor subunits (GluR5/6/7) resulted in either no labeling or very weak staining of axons (Good et al., 1993; Huntley et al., 1993; Diano et al., 1998). It is important to note that these earlier studies utilized antibodies that did not permit the individual and specific identification of the GluK1 subunit. Consequently, the previous conclusion that there was little or no definitive staining in presynaptic terminals may warrant reevaluation. Our study found significant GluK1 allocation in axonal fibers and terminals, aligning with latest research that demonstrates the role of Neto auxiliary proteins in regulating presynaptic kainate receptors and promoting the axonal recruitment of GluK1, thereby contributing to synaptic connectivity (Vesikansa et al., 2012; Orav et al., 2017).

Comparing wild-type and GASH/Sal hamsters, we observed significant differences in GluK1 distribution within crucial nuclei of the seizure neuronal network in the GASH/Sal model. Consistently with our findings, the presence of the GluK1 subunit has been previously documented in the cerebellar cortex, including within the granule and Purkinje cell layers (Bettler et al., 1990). In the GASH/Sal cerebellum, we observed a significant reduction in GluK1 protein levels and the corresponding immunoreactivity, particularly in the axons of cerebellar granule cells that form parallel fibers. This reduction implies an imbalance in excitatory signaling, leading to hyperexcitability and a higher likelihood of synchronous firing of neurons within the cerebellum. These observations lend support to the idea that the cerebellum actively participates in seizure events, rather than solely reflecting the motor aspects of seizures (Streng and Krook-Magnuson, 2021). Notably, one of the most striking distinctions in GluK1 distribution between wild-type and GASH/Sal hamsters was evident in the IC. The IC, with its distinct subdivisions, plays a critical role in audiogenic seizure activity, with the central subdivision involved in seizure initiation and the external/dorsal cortices contributing to seizure propagation (Ross and Coleman, 2000). Therefore, our findings, which reveal variations in GluK1 distribution between the central and dorsal/external regions of the IC, may correlate to the specific roles of these subdivisions in the mechanisms of audiogenic seizures in the GASH/Sal model. Our consistent results across the three employed methodological approaches (gene expression analysis, protein levels assessment, and immunohistochemistry) further support the notion that alterations in GluK1 receptors persist in the superior colliculus. This outcome is logically aligned with the interconnection of these two collicular structures and their role in audiogenic seizures (Garcia-Cairasco et al., 1993; Faingold, 1999, 2012; Ross and Coleman, 2000). The hippocampal formation is another structure closely associated with epileptogenicity (Reid et al., 1983; Ben-Ari, 1985) and the presence of GluK1 receptors, capable of co-assembling with other kainate receptor subunits in hippocampal neurons, suggests a previously underestimated diversity of these receptors and an enhanced functional complexity in this region (Paternain et al., 2000). In the GASH/Sal model, we observed a significant reduction in GluK1-immunoreactivity within the hippocampal regions, a finding that correlates with the decreased expression of the *Grik1* gene in this structure. The interpretation of these results is challenging, given the lack of consensus among studies focusing on the hippocampus and the role of GluK1 receptors in seizures. Some researchers showed that selective agonists of kainate receptors containing GluK1 induce seizure activity in preclinical models of epilepsy and observed that GluK1 knock-out mice exhibited reduced thresholds for behavioral arrest and clonic seizures (Fritsch et al., 2014). In contrast, Smolders et al. (2002) and Figueiredo et al. (2011) have shown that GluK1 antagonists inhibit seizure activity in various *in vitro* and *in vivo* models. Consequently, some authors have suggested that seizure protection can be achieved through inhibition of GluK1 receptors (Smolders et al., 2002; Figueiredo et al., 2011), while others propose that activating these receptors represents a promising antiepileptic strategy (Khalilov et al., 2002). In any case, genetically audiogenic seizure models are increasingly considered as prospective models for human temporal lobe epilepsy (Poletaeva et al., 2017; Kulikov et al., 2022), and hence, further investigation of the GASH/Sal hippocampus, particularly following audiogenic seizure stimulation, is warranted. The prefrontal cortex is involved in the generalized seizures of various genetic rodent models, in which a decline of histamine receptors has been reported in the lateral and medial prefrontal regions in the prefrontal cortex as well as the insular and cingulate cortices (Midzyanovskaya et al., 2022). However, as far as we are aware, there are no studies focused on GluK1 receptors in this context. Our results indicated GASH/Sal animals exhibited altered GluK1-immunoreactivity in the motor and cingulate cortices as well as the isidium griseum. The marked reduced immunoreactivity in the isidium griseum, a commissural hippocampal pathway that is targeted during palliative epilepsy surgery (Fauser and Zentner, 2012), might suggest a declined GluK1 receptor activity that can lead to an increase in glutamate release, which in turn enhance neuronal excitability in the afferent nuclei. An important observation from our image analysis in the prefrontal cortex was the concentrated presence of GluK1-immunoreactivity in close proximity to the cell nucleus. This peculiar subcellular distribution of GluK1 was consistently observed in the other brain areas such as the IC and hippocampus, and this pattern was more frequently seen in the GASH/Sal group compared to the wild type. The proper surface expression and synaptic trafficking of the GluK1 receptor depend on the presence of auxiliary Neto proteins (Sheng et al., 2015). Additionally, the signal peptide of GluK1 interacts directly with the ATD to inhibit the synaptic and surface expression of GluK1 (Duan et al., 2018). Considering the established influence of the extracellular ATD on the trafficking properties of GluK1 (Duan et al., 2018), it is plausible to suggest that the p.H289Y polymorphism within this domain could potentially affect the trafficking mechanism of GluK1 in the GASH/Sal model. Different isoforms of the GluK1 receptor (GluK1a, GluK1b, and GluK1c) have distinct patterns of cellular localization. While GluK1a and GluK1b can be found at the plasma membrane, the majority of GluK1c subunits predominantly remain confined in the endoplasmic reticulum (reviewed in Pahl et al., 2014). The predominant localization of GluK1-immunolabeling near the cell nucleus in the GASH/Sal model suggests that the p.H289Y polymorphism may disrupt the normal subcellular trafficking of GluK1, potentially contributing to the overall altered distribution of GluK1 protein in the GASH/Sal brain. In line with this observation, our research group concurrently conducted a functional analysis to assess the impact of the single-point mutation p.H289Y on GluK1 receptors through their expression in *Xenopus laevis* oocytes (Díaz-Rodríguez et al., 2023). Using confocal immunofluorescence microscopy to examine the distribution of GluK1 receptors within the oocytes and the two-electrode voltage-clamp technique to evaluate the mutation’s functional implications, Díaz-Rodríguez et al. (2023) found that this genetic variant potentially modifies the trafficking mechanism of GluK1, leading to increased expression and integration of mutated receptors into the oocyte membrane. Additionally, the study noted that the p.H289Y polymorphism enhances kainate-evoked currents without substantially altering the functional properties of the GluK1 receptor (Díaz-Rodríguez et al., 2023).

In summary, our study reinforces the substantial body of evidence implicating GluK1 receptors in the mechanisms underlying seizures. Human genetic studies involving families affected by idiopathic juvenile absence epilepsy have revealed elevated levels of *Grik1* polymorphisms (Sander et al., 1997). Moreover, patients with temporal lobe epilepsy exhibit modifications in GluK1 subunit expression, thereby impacting the kainate receptor function (Li et al., 2010). Rat models subjected to kainic acid-induced status epilepticus have documented disturbances in mRNA transcripts and concurrent alterations in GluK1 protein levels (Ullal et al., 2005). Notably, pharmacological investigations, particularly in the development of novel antiseizure medications, have increasingly emphasized the importance of GluK1 receptors. For instance, the anticonvulsant properties of the well-established antiepileptic drug topiramate are partly attributed to GluK1 receptor blockade (Kaminski et al., 2004). Additionally, it has been demonstrated that GluK1 receptor agonists can induce clonic seizures (Rogawski et al., 2003; Kaminski et al., 2004). Consequently, our study offers pivotal insights into the widespread alterations of GluK1 in various anatomical regions of the GASH/Sal model. This information is of paramount importance, as it lays the foundation for future experiments involving the administration of agonists or antagonists targeting kainate receptors containing GluK1 subunits. These investigations are indispensable for evaluating the potential antiseizure efficacy of GluK1 as a therapeutic target in epilepsy research.

## Data availability statement

The original contributions presented in the study are included in the article/Supplementary material, further inquiries can be directed to the corresponding author.

## Ethics statement

The animal study was approved by the Bioethics Committee of the University of Salamanca (approval number 375). The study was conducted in accordance with the local legislation and institutional requirements.

## Author contributions

SD-R: Data curation, Formal analysis, Investigation, Methodology, Visualization, Writing – review and editing. MH-T: Formal analysis, Investigation, Writing – review and editing. CG-P: Formal analysis, Investigation, Methodology, Software, Visualization, Writing – review and editing. RG-N: Conceptualization, Data curation, Formal analysis, Funding acquisition, Investigation, Methodology, Project administration, Resources, Supervision, Validation, Visualization, Writing – original draft, Writing – review and editing.

## Footnotes

1
http://www.ncbi.nlm.nih.gov/protein/

2
http://www.jalview.org/

## Supplementary material

Supplementary material 1 Topology of the GluK1 subunit, conserved epitope in GluK1 isoforms and sequence identity. **(A)** Scheme showing the topology of the GluK1 protein. The GluK1 subunit contains an extracellular amino-terminal domain (ATD) followed by a transmembrane domain (M1), a “p-loop” that dips in the lipid bilayer and forms the pore (M2), two successive transmembrane domains (M3 and M4) that delineate an extracellular loop, and an intracellular C-terminal domain (CTD). The glutamate-binding site is composed of two apposed segments (S1 and S2) in the ATD and the extracellular loop. The ATD of the kainate receptor have bi-lobed clamshell-like architectures that are composed of two regulatory domains, named R1 and R2 in upper and lower lobe, respectively. The location of the p.H289Y polymorphism in the GASH/Sal model is in the ATD-R2 domains (depicted with a red asterisk). Scheme based in the information provided in Furukawa (2012) and Selvakumar et al. (2021). **(B)** Representation of the amino acid sequences encoded by the eight identified *Grik1* isoforms in the golden hamster (*M. auratus*, GenBank GCA_017639785.1). The conserved regions between the isoforms are depicted in blue-lilac color. The region shaded in red represents the location of the p.H289Y polymorphism in the ATD and the region shaded in green represents the epitope specifically recognized by the antibody against GluK1 used in this study (see the consensus amino acid sequence at the position 380–430 amino acids). **(C)** Multiple sequence alignment of the *Grik1* isoforms that corresponds to the region shaded in red [position 280–298 amino acids in panel **(B)**], in which the single nucleotide polymorphism (p.His289Tyr; red asterisk) was identified. The longest isoform (identified as canonical) was used as the consensus sequence. Level of each amino acid conservation is represented underneath as a bar chart, using Clustalx coloring (Jalview program 2.11.2 version; http://www.jalview.org/). The conserved columns with a score of 11 are indicated by “χ” (score of 11 with default amino acid property grouping), and columns with a score of 10 have mutations but all properties are conserved are marked with a “ + .” **(D)** Multiple sequence alignment of GluK1 (at the positions 281–297 amino acids) in the golden hamster, mouse, rat, and human. Identity values (%) correspond to full-length sequences. Notice that the mutation site (indicated by a red asterisk) is conserved across species.

Supplementary material 2 A video showcasing the dynamic rotation of the ribbon representation of the monomeric form of the GluK1 subunit. The 3D protein structures, predicted by AlphaFold, correspond to both the wild-type protein structure and the mutated form (histidine-to-tyrosine substitution at the position 289) found in the GASH/Sal model. This video corresponds to the results depicted in Figure 2.

Supplementary material 3 Raw data of qPCR used for analyses. The qPCR data included a set of 5 biological replicates (sample cases) for each experimental group (wild-type and GASH/Sal animals), triplicate technical replicates as well as the Ct values of housekeeping genes (β-actin and tubulin) used for data normalization. Abbreviations of genes: *Grik1* (glutamate receptor ionotropic kainate-1); *Actb* (β-actin); *Tbp* (tubulin).

Supplementary material 4 Animated video showing 3D renderings of the maximum projection of the confocal stacks depicting the GluK1-immunoreactivity in the inferior colliculus of the wild-type and GASH/Sal hamsters (video corresponding to Figures 7Ae, Be, respectively).

Supplementary material 5 Confocal micrographs showing Gluk1-immunolabeling in the external cortex of the GASH/Sal inferior colliculus. Orthogonal view from different planes (x/y, x/z or y/z) of the confocal images illustrate GluK1-immunolabeling concentrated around the DAPI-stained nucleus. Scale bar = 10 μm.

Supplementary material 6 Animated video showing 3D renderings of the maximum projection of the confocal stacks depicting the GluK1-immunoreactivity in the superior colliculus of the wild-type hamsters (video corresponding to Figure 8Ae).

Supplementary material 7 Bright field and confocal micrographs showing Gluk1-immunolabeling in the dentate gyrus of the wild-type and GASH/Sal hamsters. Notice the remarkable reduction of GluK1-punctate immunostaining (arrowheads) in the GASH/Sal hamster as compared to the wild-type animal. The maximum intensity projections of confocal Z-stack immunofluorescence images (depicted in lower part of the panel) show GluK1 (green), NeuN (red), and DAPI staining of DNA (blue). Scale bars = 10 and 5 μm for bright field and confocal images, respectively.

Supplementary material 8 Confocal micrographs depicting Gluk1-immunolabeling in the dentate gyrus of the wild-type hamster brain. Maximum intensity projections of confocal Z-stack images show an axonal fiber immunostained for GluK1 (arrows). The panels below show higher magnification in separated channels GluK1 (green), NeuN (red), and DAPI staining of DNA (blue). Scale bars = 5 μm in the upper panel and 2 μm for lower panels.

Supplementary material 9 Confocal micrographs showing Gluk1-immunolabeling in the dentate gyrus of the GASH/Sal brain. Orthogonal view from different planes (x/y, x/z or y/z) of the confocal images illustrate GluK1-immunolabeling concentrated around the DAPI-stained nucleus. Scale bar = 5 μm.

Supplementary material 10 Animated video showing 3D renderings of the maximum projection of the confocal stacks, highlighting the GluK1-immunoreactivity in the layer I-II of the cingulate cortex in wild-type hamsters (video corresponding to Figure 10Ac).

Supplementary material 11 Animated video of the maximum projection of the confocal stacks shows details of GluK1-immunoreactivity in cells of the medial prefrontal cortex (prelimbic area) in the wild-type and GASH/Sal hamsters. Notice reduced punctate GluK1-immunostaining in cell bodies in the GASH/Sal as compared to the control animal.

Supplementary material 12 Confocal micrographs showing Gluk1-immunolabeling in the medial prefrontal cortex (cingulate cortex) of the GASH/Sal brain. Orthogonal view from different planes (x/y, x/z or y/z) of the confocal images illustrate GluK1-immunolabeling concentrated around the DAPI-stained nucleus of the neuron depicted with a magenta arrowhead in Figure 10Bc. Scale bar = 5 μm.
