# Supplementary material for: Delving into the significance of the His289Tyr single-nucleotide polymorphism in the glutamate ionotropic receptor kainate-1 (Grik1) gene of a genetically audiogenic seizure model
Source: Front Mol Neurosci. 2024 Jan 5;16:1322750. doi: 10.3389/fnmol.2023.1322750 (PMC10797026; doi:10.3389/fnmol.2023.1322750)
Supplement: Supplementary material 1 — Topology of the GluK1 subunit, conserved epitope in GluK1 isoforms and sequence identity. (A) Scheme showing the topology of the GluK1 protein. The GluK1 subunit contains an extracellular amino-terminal domain (ATD) followed by a transmembrane domain (M1), a “p-loop” that dips in the lipid bilayer and forms the pore (M2), two successive transmembrane domains (M3 and M4) that delineate an extracellular loop, and an intracellular C-terminal domain (CTD). The glutamate-binding site is composed of two apposed segments (S1 and S2) in the ATD and the extracellular loop. The ATD of the kainate receptor have bi-lobed clamshell-like architectures that are composed of two regulatory domains, named R1 and R2 in upper and lower lobe, respectively. The location of the p.H289Y polymorphism in the GASH/Sal model is in the ATD-R2 domains (depicted with a red asterisk). Scheme based in the information provided in Furukawa (2012) and Selvakumar et al. (2021). (B) Representation of the amino acid sequences encoded by the eight identified Grik1 isoforms in the golden hamster (M. auratus, GenBank GCA_017639785.1). The conserved regions between the isoforms are depicted in blue-lilac color. The region shaded in red represents the location of the p.H289Y polymorphism in the ATD and the region shaded in green represents the epitope specifically recognized by the antibody against GluK1 used in this study (see the consensus amino acid sequence at the position 380–430 amino acids). (C) Multiple sequence alignment of the Grik1 isoforms that corresponds to the region shaded in red [position 280–298 amino acids in panel (B)], in which the single nucleotide polymorphism (p.His289Tyr; red asterisk) was identified. The longest isoform (identified as canonical) was used as the consensus sequence. Level of each amino acid conservation is represented underneath as a bar chart, using Clustalx coloring (Jalview program 2.11.2 version; http://www.jalview.org/). The conserved columns with a score of 11 ar [file Data_Sheet_1.zip › Supplementary Material 3.pdf]

Raw data of RT-qPCR used for analyses (Ct values)

Animal group		Cerebellum			Inferior colliculus			Superior colliculus			Hippocampus			Prefrontol Cortex		
	**Animal ID**	***Grik1***	***Actb***	***Tbp***	***Grik1***	***Actb***	***Tbp***	***Grik1***	***Actb***	***Tbp***	***Grik1***	***Actb***	***Tbp***	***Grik1***	***Actb***	***Tbp***
**CONTROL (Wild Type)**	**16706**	20,843	20,124	21,982	25,504	19,644	24,112	24,336	19,082	24,444	24,595	17,887	23,846	25,937	19,059	23,231
		20,828	20,386	21,803	25,790	21,312	24,183	25,137	19,450	24,944	24,696	16,244	23,751	25,897	19,593	23,336
		20,755	20,174	21,903	25,559	19,787	24,506	24,823	19,018	24,589	24,880	17,574	23,609	26,365	19,413	23,002
	**16707**	21,249	20,235	21,509	25,214	19,956	24,181	23,377	19,233	24,325	24,576	18,599	24,708	24,495	18,773	23,847
		21,212	20,204	21,674	25,086	19,605	24,306	23,562	19,212	24,194	24,499	18,210	24,224	24,526	19,224	24,771
		21,245	20,246	22,403	24,845	18,225	24,328	23,486	19,345	23,977	24,472	18,329	24,166	25,059	19,698	24,418
	**16708**	21,338	20,834	21,279	24,816	19,473	23,451	23,256	19,140	23,960	24,455	17,891	26,542	24,448	19,068	23,340
		21,651	20,903	21,654	24,969	19,005	23,554	23,577	19,124	23,976	25,091	17,747	25,312	24,581	18,906	23,091
		20,460	20,784	21,350	24,551	18,946	23,640	23,561	19,480	23,985	24,865	18,054	24,275	24,974	18,835	23,705
	**16709**	21,901	20,014	21,326	24,977	19,455	23,963	24,595	19,511	24,872	24,080	17,286	24,182	23,284	17,793	22,165
		21,982	19,939	21,313	24,787	19,196	23,718	24,782	19,754	24,276	23,614	17,169	24,347	23,303	17,687	22,155
		21,659	19,930	21,245	25,055	19,243	23,682	24,578	19,705	23,764	23,849	17,250	23,882	23,311	17,744	22,137
	**16710**	21,524	20,401	21,374	26,095	19,637	Undetermined*	23,950	Undetermined*	23,521	23,848	17,982	24,729	24,764	19,941	24,441
		21,521	20,694	21,631	25,977	19,712	Undetermined*	24,491	20,722	23,728	23,864	17,722	24,606	24,312	20,585	24,347
		21,461	20,427	21,713	26,047	Undetermined*	Undetermined*	23,836	20,852	23,723	23,927	17,809	25,205	24,390	Undetermined*	24,134
**GASH/Sal**	**4567**	23,212	22,928	24,139	27,280	22,380	27,305	23,567	19,696	23,930	24,210	16,423	23,261	24,779	18,517	24,093
		22,817	22,976	24,725	27,265	22,503	27,226	23,566	19,532	24,351	24,252	16,230	23,176	24,886	17,964	23,534
		22,815	22,838	24,571	27,349	22,356	27,837	23,573	19,788	24,339	23,971	16,554	22,775	25,122	19,591	23,532
	**4568**	22,543	22,411	25,784	23,970	18,660	24,595	23,997	21,459	25,323	24,094	17,739	23,532	24,617	19,405	24,029
		22,233	22,427	25,709	24,161	18,422	24,460	23,960	21,115	24,312	24,686	17,887	22,968	24,503	19,410	24,161
		25,989	22,639	25,517	23,944	18,921	24,453	24,039	21,171	25,455	24,786	17,676	23,096	24,846	19,425	23,211
	**4569**	21,596	21,688	25,266	24,471	19,764	Undetermined*	24,912	20,046	24,486	24,981	17,342	24,223	24,942	19,084	23,544
		21,910	21,532	25,106	24,882	19,729	27,251	24,279	20,010	24,444	25,423	17,871	24,388	25,563	19,094	25,613
		21,556	20,554	25,099	24,671	20,092	26,913	24,748	20,183	24,374	24,728	Undetermined*	24,567	24,622	19,003	23,972
	**4580**	21,144	21,191	24,798	24,278	19,077	25,982	23,327	20,416	24,977	28,907	18,777	23,786	24,751	18,608	24,219
		21,363	21,225	24,183	24,363	19,156	26,199	24,178	20,363	25,536	24,713	17,322	23,900	24,720	18,529	24,485
		21,393	21,274	24,381	24,416	Undetermined*	25,834	24,421	20,506	25,499	25,815	19,007	23,668	24,545	18,464	24,698
	**4583**	20,625	21,410	24,336	24,879	19,297	Undetermined*	23,970	20,501	24,771	24,745	17,268	23,982	24,762	20,059	24,126
		21,092	21,727	24,163	24,256	19,298	Undetermined*	23,216	20,566	24,405	24,572	17,588	24,132	24,549	20,042	24,273
		21,382	21,577	24,326	24,869	Undetermined*	Undetermined*	23,980	20,623	24,764	24,796	Undetermined*	23,809	24,848	19,967	24,513

* Outlier and undetermined values (below the detection limit of the RT-qPCR assay) of biological replicates were not included in the data sheet.

Abbreviation of Genes: *Grik1 (glutamate receptor ionotropic kainate-1); Actb* (β-actin); *Tbp* (tubulin)
