# Supplementary figures and images for: Delving into the significance of the His289Tyr single-nucleotide polymorphism in the glutamate ionotropic receptor kainate-1 (Grik1) gene of a genetically audiogenic seizure model
Source: Front Mol Neurosci. 2024 Jan 5;16:1322750. doi: 10.3389/fnmol.2023.1322750 (PMC10797026; doi:10.3389/fnmol.2023.1322750)

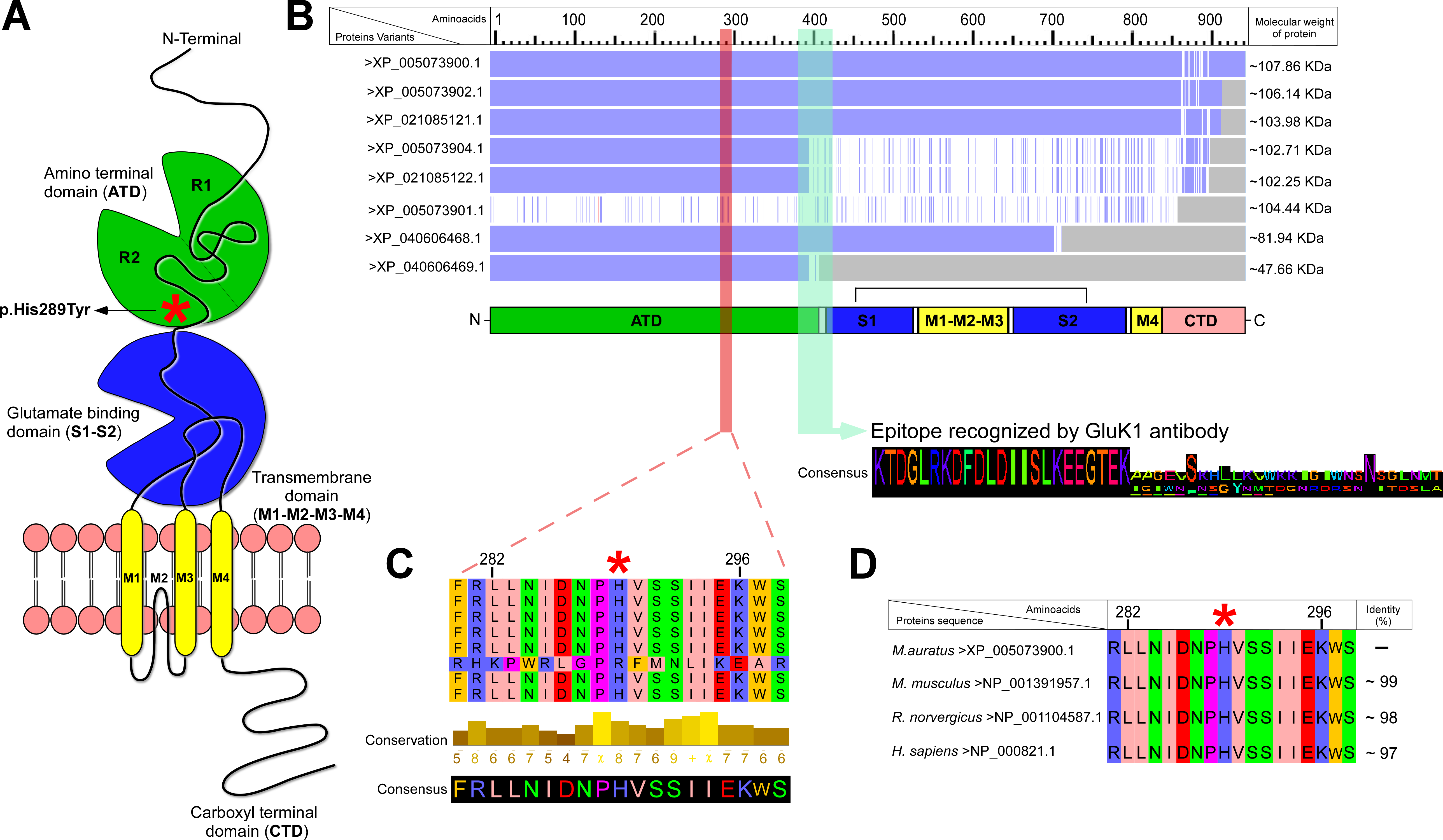

Supplement: Supplementary material 1 — Topology of the GluK1 subunit, conserved epitope in GluK1 isoforms and sequence identity. (A) Scheme showing the topology of the GluK1 protein. The GluK1 subunit contains an extracellular amino-terminal domain (ATD) followed by a transmembrane domain (M1), a “p-loop” that dips in the lipid bilayer and forms the pore (M2), two successive transmembrane domains (M3 and M4) that delineate an extracellular loop, and an intracellular C-terminal domain (CTD). The glutamate-binding site is composed of two apposed segments (S1 and S2) in the ATD and the extracellular loop. The ATD of the kainate receptor have bi-lobed clamshell-like architectures that are composed of two regulatory domains, named R1 and R2 in upper and lower lobe, respectively. The location of the p.H289Y polymorphism in the GASH/Sal model is in the ATD-R2 domains (depicted with a red asterisk). Scheme based in the information provided in Furukawa (2012) and Selvakumar et al. (2021). (B) Representation of the amino acid sequences encoded by the eight identified Grik1 isoforms in the golden hamster (M. auratus, GenBank GCA_017639785.1). The conserved regions between the isoforms are depicted in blue-lilac color. The region shaded in red represents the location of the p.H289Y polymorphism in the ATD and the region shaded in green represents the epitope specifically recognized by the antibody against GluK1 used in this study (see the consensus amino acid sequence at the position 380–430 amino acids). (C) Multiple sequence alignment of the Grik1 isoforms that corresponds to the region shaded in red [position 280–298 amino acids in panel (B)], in which the single nucleotide polymorphism (p.His289Tyr; red asterisk) was identified. The longest isoform (identified as canonical) was used as the consensus sequence. Level of each amino acid conservation is represented underneath as a bar chart, using Clustalx coloring (Jalview program 2.11.2 version; http://www.jalview.org/). The conserved columns with a score of 11 ar [file Data_Sheet_1.zip › Supplementary Material 1.tif]

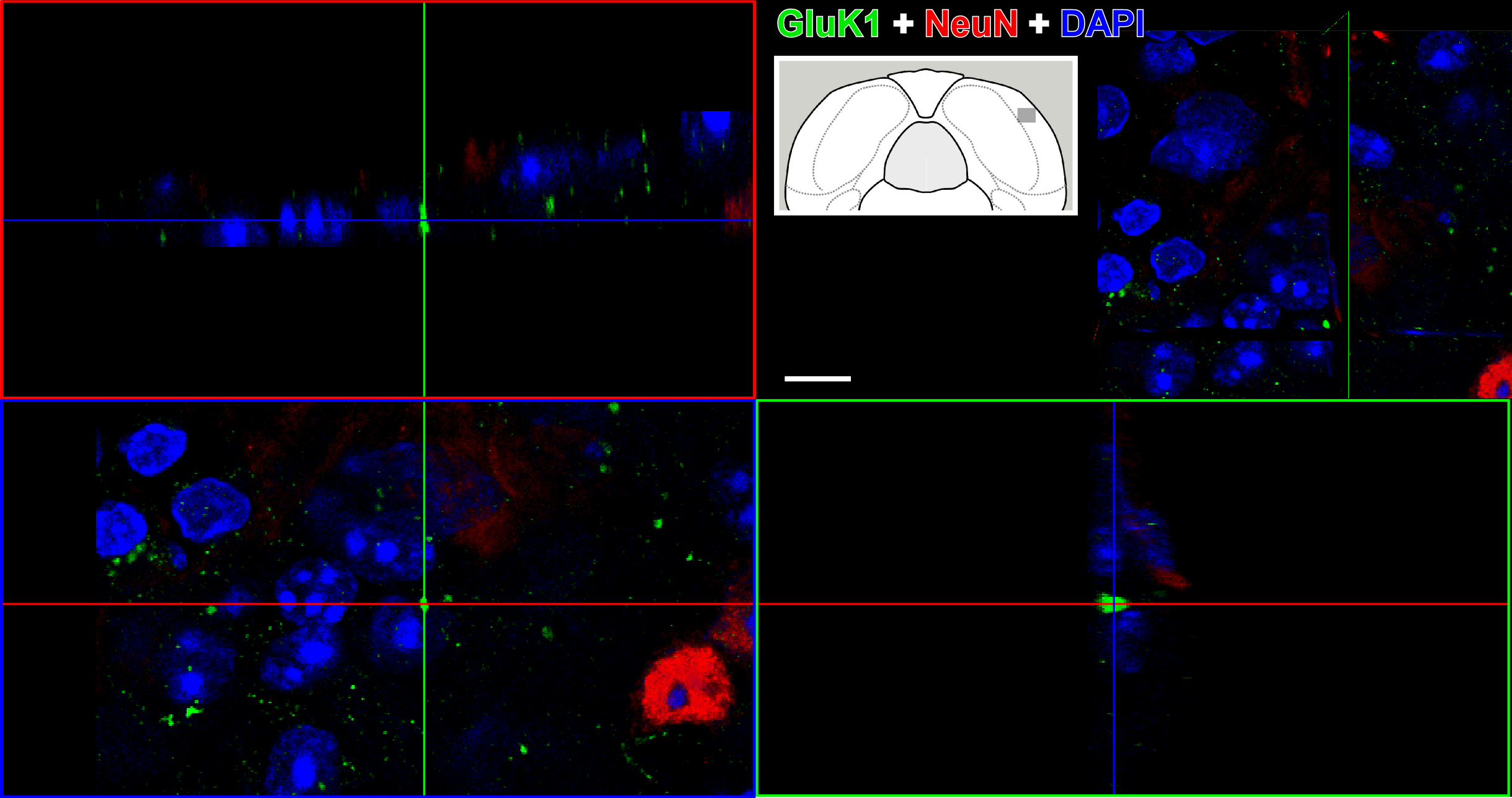

Supplement: Supplementary material 1 — Topology of the GluK1 subunit, conserved epitope in GluK1 isoforms and sequence identity. (A) Scheme showing the topology of the GluK1 protein. The GluK1 subunit contains an extracellular amino-terminal domain (ATD) followed by a transmembrane domain (M1), a “p-loop” that dips in the lipid bilayer and forms the pore (M2), two successive transmembrane domains (M3 and M4) that delineate an extracellular loop, and an intracellular C-terminal domain (CTD). The glutamate-binding site is composed of two apposed segments (S1 and S2) in the ATD and the extracellular loop. The ATD of the kainate receptor have bi-lobed clamshell-like architectures that are composed of two regulatory domains, named R1 and R2 in upper and lower lobe, respectively. The location of the p.H289Y polymorphism in the GASH/Sal model is in the ATD-R2 domains (depicted with a red asterisk). Scheme based in the information provided in Furukawa (2012) and Selvakumar et al. (2021). (B) Representation of the amino acid sequences encoded by the eight identified Grik1 isoforms in the golden hamster (M. auratus, GenBank GCA_017639785.1). The conserved regions between the isoforms are depicted in blue-lilac color. The region shaded in red represents the location of the p.H289Y polymorphism in the ATD and the region shaded in green represents the epitope specifically recognized by the antibody against GluK1 used in this study (see the consensus amino acid sequence at the position 380–430 amino acids). (C) Multiple sequence alignment of the Grik1 isoforms that corresponds to the region shaded in red [position 280–298 amino acids in panel (B)], in which the single nucleotide polymorphism (p.His289Tyr; red asterisk) was identified. The longest isoform (identified as canonical) was used as the consensus sequence. Level of each amino acid conservation is represented underneath as a bar chart, using Clustalx coloring (Jalview program 2.11.2 version; http://www.jalview.org/). The conserved columns with a score of 11 ar [file Data_Sheet_1.zip › Supplementary Material 5.tif]

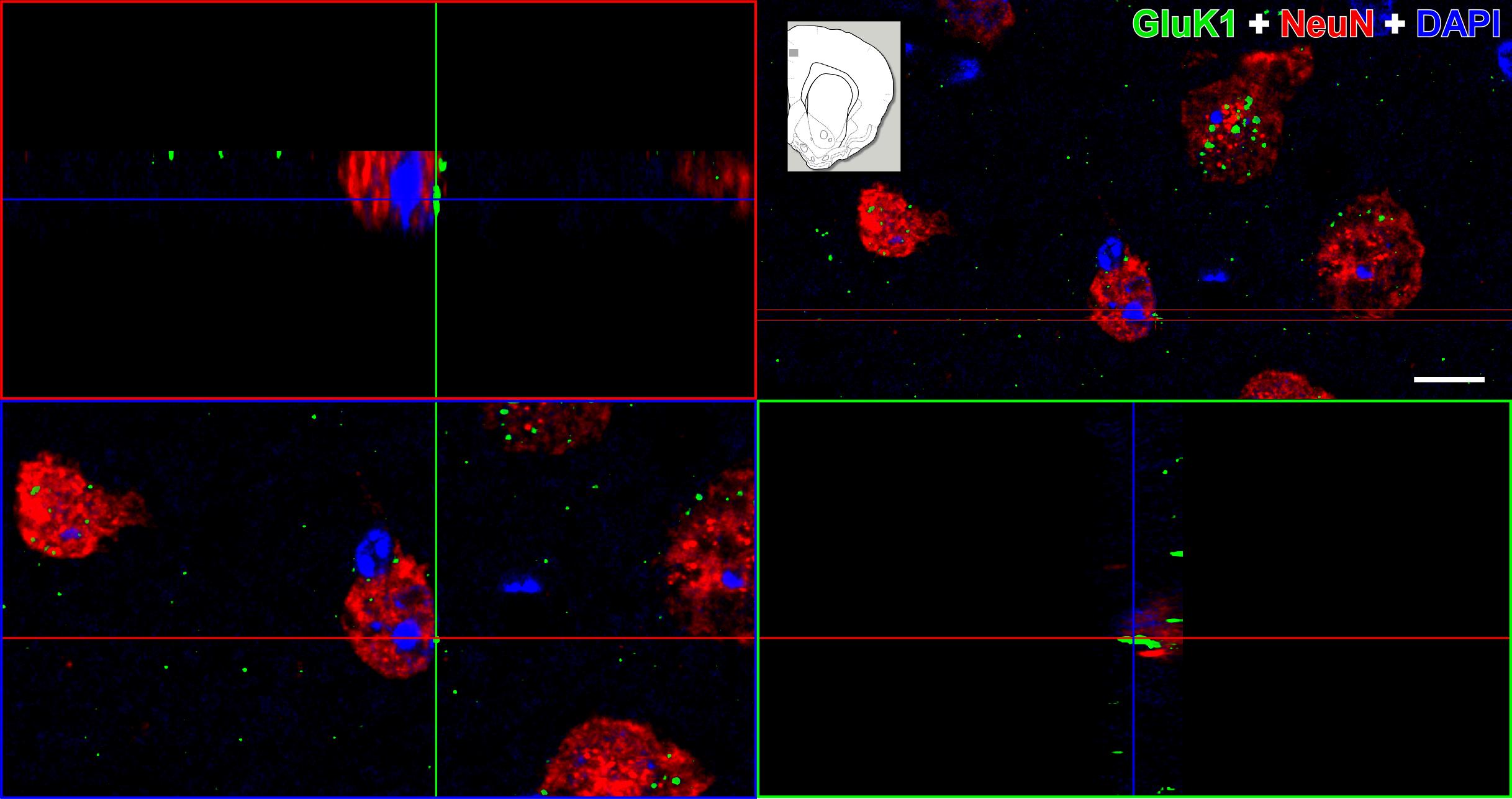

Supplement: Supplementary material 7 — Bright field and confocal micrographs showing Gluk1-immunolabeling in the dentate gyrus of the wild-type and GASH/Sal hamsters. Notice the remarkable reduction of GluK1-punctate immunostaining (arrowheads) in the GASH/Sal hamster as compared to the wild-type animal. The maximum intensity projections of confocal Z-stack immunofluorescence images (depicted in lower part of the panel) show GluK1 (green), NeuN (red), and DAPI staining of DNA (blue). Scale bars = 10 and 5 μm for bright field and confocal images, respectively. [file Data_Sheet_2.zip › Supplementary Material 12.tif]

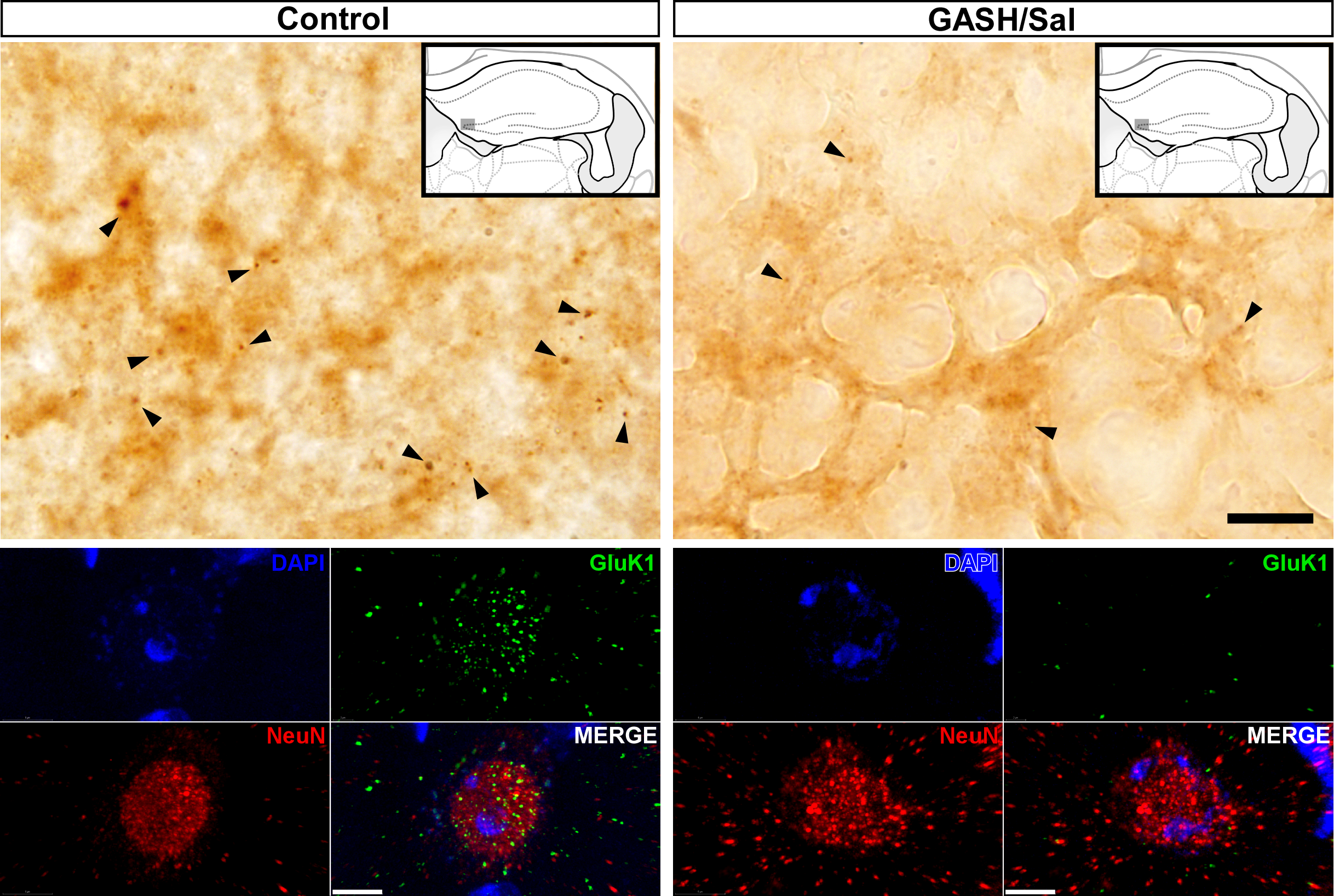

Supplement: Supplementary material 7 — Bright field and confocal micrographs showing Gluk1-immunolabeling in the dentate gyrus of the wild-type and GASH/Sal hamsters. Notice the remarkable reduction of GluK1-punctate immunostaining (arrowheads) in the GASH/Sal hamster as compared to the wild-type animal. The maximum intensity projections of confocal Z-stack immunofluorescence images (depicted in lower part of the panel) show GluK1 (green), NeuN (red), and DAPI staining of DNA (blue). Scale bars = 10 and 5 μm for bright field and confocal images, respectively. [file Data_Sheet_2.zip › Supplementary Material 7.tiff]

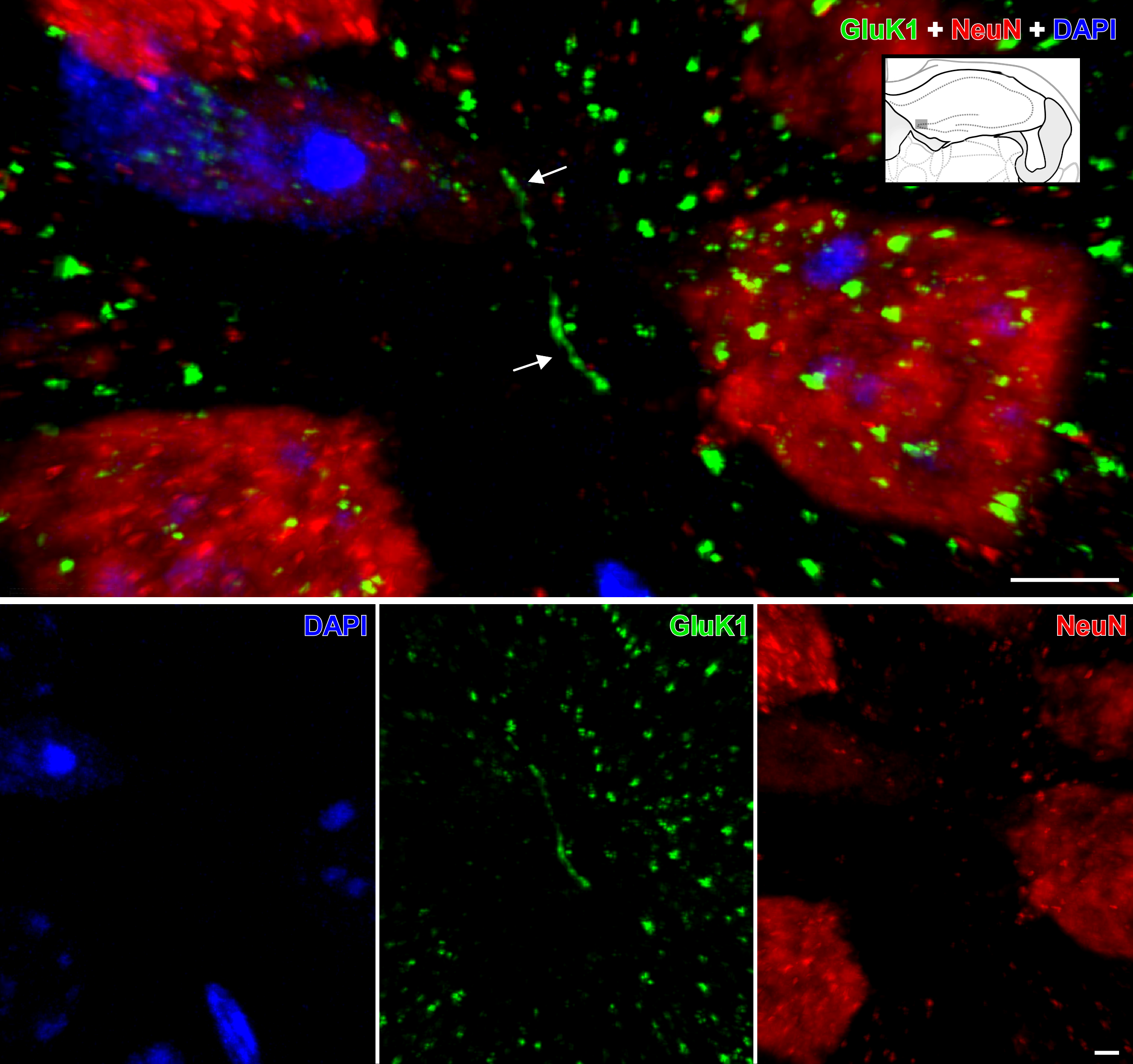

Supplement: Supplementary material 7 — Bright field and confocal micrographs showing Gluk1-immunolabeling in the dentate gyrus of the wild-type and GASH/Sal hamsters. Notice the remarkable reduction of GluK1-punctate immunostaining (arrowheads) in the GASH/Sal hamster as compared to the wild-type animal. The maximum intensity projections of confocal Z-stack immunofluorescence images (depicted in lower part of the panel) show GluK1 (green), NeuN (red), and DAPI staining of DNA (blue). Scale bars = 10 and 5 μm for bright field and confocal images, respectively. [file Data_Sheet_2.zip › Supplementary Material 8.tif]

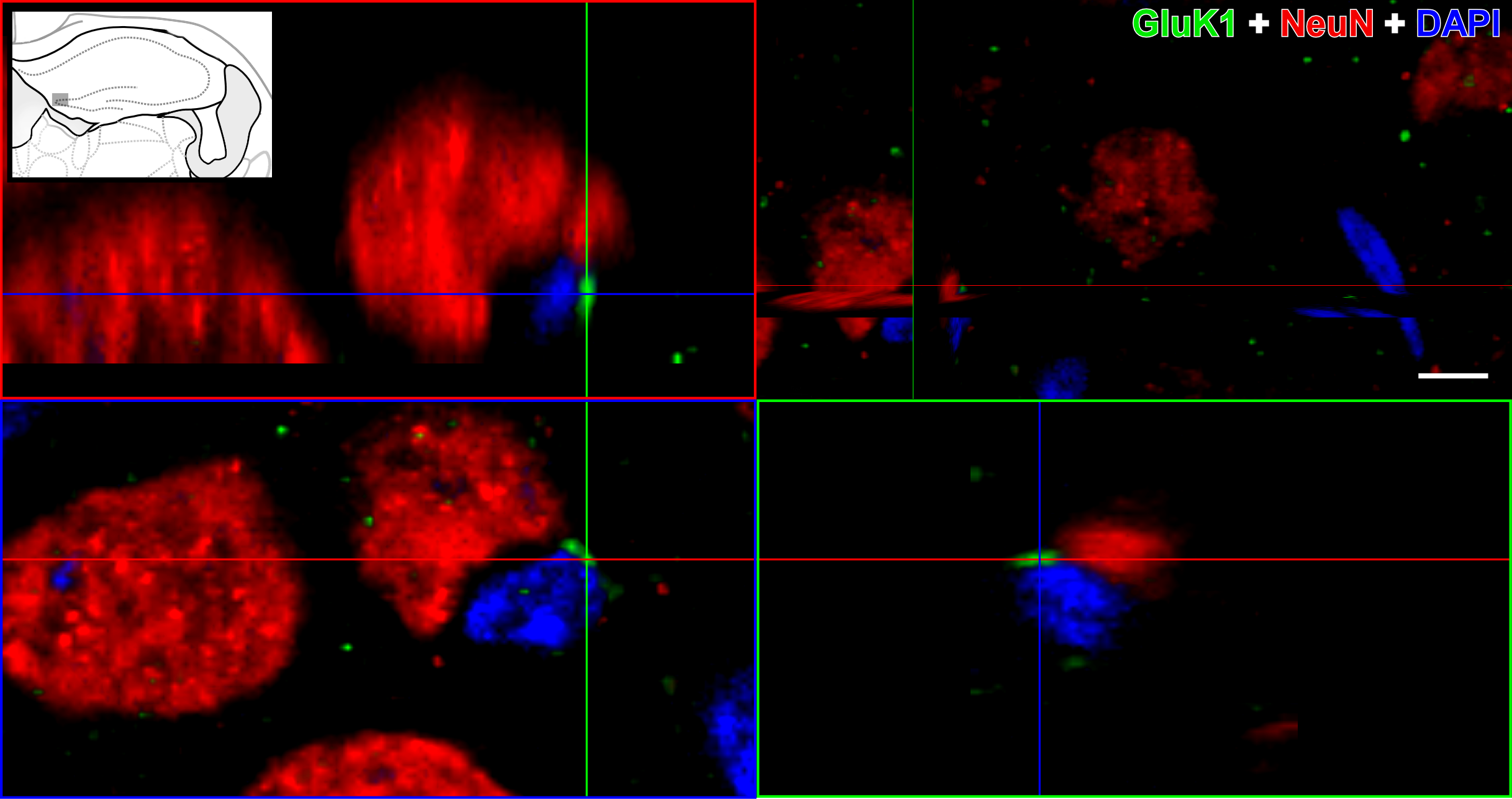

Supplement: Supplementary material 7 — Bright field and confocal micrographs showing Gluk1-immunolabeling in the dentate gyrus of the wild-type and GASH/Sal hamsters. Notice the remarkable reduction of GluK1-punctate immunostaining (arrowheads) in the GASH/Sal hamster as compared to the wild-type animal. The maximum intensity projections of confocal Z-stack immunofluorescence images (depicted in lower part of the panel) show GluK1 (green), NeuN (red), and DAPI staining of DNA (blue). Scale bars = 10 and 5 μm for bright field and confocal images, respectively. [file Data_Sheet_2.zip › Supplementary Material 9.tif]
